# Gene Expression and Proteomics Studies Suggest an Involvement of Multiple Pathways Under Day and Day–Night Combined Heat Stresses During Grain Filling in Wheat

**DOI:** 10.3389/fpls.2021.660446

**Published:** 2021-05-31

**Authors:** Venkatesh Chunduri, Amandeep Kaur, Shubhpreet Kaur, Aman Kumar, Saloni Sharma, Natasha Sharma, Pargat Singh, Payal Kapoor, Satveer Kaur, Anita Kumari, Joy Roy, Jaspreet Kaur, Monika Garg

**Affiliations:** ^1^Agri-Biotechnology Division, National Agri-Food Biotechnology Institute, Mohali, India; ^2^Department of Biotechnology Engineering, University Institute of Engineering and Technology, Panjab University, Chandigarh, India; ^3^Department of Immunopathology, Post Graduate Institute of Medical and Education and Research, Chandigarh, India; ^4^School of Agricultural Biotechnology, Punjab Agricultural University, Ludhiana, India

**Keywords:** heat stress, wheat grain, gene expression, 2D-PAGE, MALDI–TOF

## Abstract

Recent weather fluctuations imposing heat stress at the time of wheat grain filling cause frequent losses in grain yield and quality. Field-based studies for understanding the effect of terminal heat stress on wheat are complicated by the effect of multiple confounding variables. In the present study, the effect of day and day–night combined heat stresses during the grain-filling stage was studied using gene expression and proteomics approaches. The gene expression analysis was performed by using real-time quantitative PCR (RT-qPCR). The expression of genes related to the starch biosynthetic pathway, starch transporters, transcription factors, and stress-responsive and storage proteins, at four different grain developmental stages, indicated the involvement of multiple pathways. Under the controlled conditions, their expression was observed until 28 days after anthesis (DAA). However, under the day stress and day–night stress, the expression of genes was initiated earlier and was observed until 14 DAA and 7 DAA, respectively. The protein profiles generated using two-dimensional polyacrylamide gel electrophoresis (2D-PAGE) and matrix-assisted laser desorption/ionization time-of-flight mass spectroscopy (MALDI-TOF MS/MS) showed a differential expression of the proteins belonging to multiple pathways that included the upregulation of proteins related to the translation, gliadins, and low-molecular-weight (LMW) glutenins and the downregulation of proteins related to the glycolysis, photosynthesis, defense, and high-molecular-weight (HMW) glutenins. Overall, the defense response to the day heat stress caused early gene expression and day–night heat stress caused suppression of gene expression by activating multiple pathways, which ultimately led to the reduction in grain-filling duration, grain weight, yield, and processing quality.

## Introduction

World population by enlarging depends on wheat as a cereal food. Despite significant improvement in wheat production achieved over the last couple of decades, wheat production is always threatened due to biotic and abiotic stress. Recent climate change has experienced frequent episodes of unexpected fluctuations in temperature. Such adverse temperatures cause severe losses, particularly during the grain-filling stage in wheat. Despite being a high-yielding cereal crop, wheat is sensitive to heat stress (Porter and Gawith, 1999). The optimum temperature at the wheat reproductive period is 18–24°C (Stone et al., 1997). The high temperatures during the plant growth and development might cause 2.5–10% of yield losses in field crops (Hatfield et al., 2011). In the case of wheat, every 1°C increase above the optimum temperature reduces the yield by 4–6% (Bennett et al., 2012; Liu et al., 2016). Many climate models on global warming had predicted that by the end of the Twenty-first century, the optimum temperature of the globe would increase up to 6°C (De Costa, 2011).

In many countries, heat stress is mainly experienced during seed development. Although all the growth stages of wheat are sensitive to high-temperature stresses, the reproductive phase is the most critical. A slight elevation in temperature during the pollination affects pollen sterility, floret abortion, damage to stigma, and fertilization (Wollenweber et al., 2003; Kumar et al., 2015; Rieu et al., 2017). The terminal heat stress reduces photosynthesis due to thylakoid membrane damage (Ristic et al., 2007) and inhibition of photosystem II (PSII) (Wollenweber et al., 2003). Heat stress during the reproductive stage also increases the senescence rate, resulting in the decrease of grain-filling duration and the subsequent decrease in the grain weight, defragmented starch granules, and seed quality (Hays et al., 2007; Kumar et al., 2013). Heat stress also causes downregulation of several genes related to various pathways. High postanthesis temperature downregulated the genes involved in the pericarp cell wall expansion leading to reduction in matured grain weight (Kino et al., 2020). Rangan et al. (2020) identified a cluster of genes that are upregulated in the heat-tolerant cultivar during early and late grain-filling heat stress as compared with the susceptible cultivar. Recent genome-wide studies have identified various heat shock protein (HSP) genes and transcription factors under heat and drought stresses (Liu et al., 2015; Kumar et al., 2020).

Most of the previous studies had addressed the effects of heat stress imposed only either during daytime (Wang et al., 2015) or nighttime (García et al., 2015; Impa et al., 2019). Besides, limited efforts are employed to reveal the molecular mechanisms explaining yield reduction in heat stress. Some studies report single time-point stress (Impa et al., 2020), while others are limited to a few hours of heat stress (Kumar et al., 2014). Only a few studies had observed the gene expression throughout the grain-filling period, but they were restricted only to the genes involved in the starch biosynthesis pathway (Hurkman et al., 2003). Although gene expression is important to investigate the heat stress response in developing an endosperm, ultimately, the protein content and composition of matured grains determine the processing quality of wheat. Recently, high-throughput proteomics approaches, such as two-dimensional gel electrophoresis/mass spectroscopy (2DE/MS) and iTRAQ, have become powerful tools for identifying and quantifying large number of differentially expressed proteins (Finnie et al., 2011; Zhang et al., 2017). In this study, the effects of day stress and day–night combined stress on gene expression at different grain-filling stages were investigated. Efforts were also being made to understand the protein profiles of the matured grains by 2D polyacrylamide gel electrophoresis (2D-PAGE) combined with the matrix-assisted laser desorption/ionization time-of-flight mass spectroscopy (MALDI-TOF MS/MS) analysis.

## Materials and Methods

### Plant Material and Growth Conditions

The current study used BWL4444 (HD2967+ Yr10), a promising heat-tolerant wheat germplasm to understand the heat stress response during the grain-filling period. The germplasm was obtained from Punjab Agricultural University, Ludhiana, Punjab, India. A total of 120 seeds were sown in 400 ml pots filled with vermiculite. Osgrel and Somerville soil-less planting media were supplied to each pot. The plants were grown under controlled conditions of 24/17°C and 16 h of light (300 μM m^−2^ s^−2^) until anthesis. Two days after anthesis (DAA), plants were divided into three sets of 40 plants each. These plants were then subjected to three temperature regimes—(1) control (24/17°C), (2) day heat stress (35/17°C), and (3) day–night heat stress (35/24°C). The heat treatment was continued until maturity. To simulate the field conditions, the temperature in the growth chambers was gradually increased at the rate of 2–3°C/h, starting at 6.00 A.M., and the highest temperature of 35°C was achieved at 11.00 A.M. The highest temperature was provided for 6 h from 11.00 A.M. to 5.00 P.M. From 5.00 P.M. to 9.00 P.M., the temperature was then gradually reduced at the rate of 2–3°C/h to 17°C or 24°C for day stress and day–night stress, respectively. The night heat treatment in the third set of plants was provided for 10 h from 9.00 P.M. to 7.00 A.M. (Supplementary Table 1).

### Biochemical Parameters

Flag leaves from more than three biological replicates were collected at 7 DAA at 5 P.M. They were immediately frozen in liquid nitrogen and stored at −80°C for biochemical analysis. Proline was quantified according to Bates et al. (1973), with slight modification. Of note, 0.2 g of fresh leaves were homogenized with 2 ml of 3% aqueous sulfosalicylic acid. Furthermore, 1 ml of acid ninhydrin solution and 1 ml of glacial acetic acid were added to 1 ml of the supernatant and incubated in a boiling water bath for 60 min. After cooling down to the room temperature, 4 ml of toluene was added to the reaction mixture and vortexed thoroughly. The absorbance of the toluene layer was measured at 520 nm using toluene as a blank.

For estimating hydrogen peroxide (H_2_O_2_), 0.2 g of fresh leaves were homogenized in 2 ml of 0.1% trichloroacetic acid (TCA) (Alexieva et al., 2001). Furthermore, 1 ml of the supernatant was mixed with 2 ml of assay mixture containing 4 mM of potassium iodide and 0.1 mM of potassium phosphate buffer (pH 7.0) and incubated at the room temperature in the dark for 1 h. The absorbance was read at 390 nm, and the concentration was quantified using the H_2_O_2_ standard curve.

Extraction of the antioxidant enzyme was carried out according to Yang et al. (2008). Of note, 200 mg of leaf tissue was homogenized in 2 ml of buffer (50 mM of potassium phosphate buffer [pH 7.0], 1 mM of EDTA, 2% polyvinyl pyrrolidone (PVP), and 0.05% Triton-X-100). The homogenate was then centrifuged at 10,000 × *g* at 4°C for 15 min. Guaiacol peroxidase (GPOX) was assayed (Simova-Stoilova et al., 2008) by mixing 50 μl of enzyme extract with 2.5 ml of buffer containing 100 mM of potassium phosphate buffer (pH 6.5), 50 mM of guaiacol, and 32 mM of H_2_O_2_. A change in absorbance at 470 nm was recorded at 25°C. A change in mM of tetraguaiacol per minute was calculated using the molar extinction coefficient of tetraguaiacol (26.6/mM/cm).

### Yield-Related Parameters

At physiological maturity, the primary tillers from each plant were harvested, and yield-related components were recorded. The kernels from each spikelet were harvested separately, and kernel length and width were measured using a vernier caliper. On an average, we took two kernels/spikelet from four different spikes for measuring kernel dimensions. Kernel weight per spike and thousand kernel weight (TKW) were also recorded.

### Differential Gene Expression

For differential gene expression, the developing grains were collected at 7, 14, 21, and 28 DAA at 5.00 P.M. and immediately frozen in liquid nitrogen and stored at −80°C. Total RNA was isolated from the developing grains using the TRIZOL® reagent (Ambion, Austin, Texas, USA). Two micrograms of DNA-free RNA were used for the first-strand cDNA synthesis using the first strand cDNA synthesis kit (ThermoFisher, Massachusetts, USA) with random hexamer primers as per the guidelines of the manufacturer. The real-time quantitative PCR (RT-qPCR) was performed for selected candidate genes involved in starch biosynthesis and transport, storage protein synthesis, stress response, and transcription factors (Supplementary Table 2) using SYBR Green Real-Time-PCR Master Mix (Ambion) on ABI 7700 sequence detector (Applied Biosystems, Foster City, CA, USA). Each reaction was carried out in three biological replicates and with three technical replicates each. Amplification was performed according to the default cycle, and fold change values were calculated based on average 2^−Δ*ΔCT*^ values with ADP-ribosylation factor (ADPRF) as an internal control (Livak and Schmittgen, 2001; Kumar et al., 2020). MeV (multiexperiment viewer) software was used for the data analysis.

### Proteomic Analysis

#### Protein Extraction and Quantification

For proteome analysis, mature grains of more than three biological replicates were collected. Protein extraction was performed sequentially, and separate fractions of albumins–globulins (0.1 M sodium chloride in 25 mM sodium phosphate buffer of pH 7.0), gliadins (1.5 M dimethylformamide), and glutenins [50 mM Tris-HCL dissolved in 50% (v/v) isopropanol] (Garg et al., 2014) were collected. The supernatants from different fractions were precipitated with four volumes of acetone containing 10% trichloroacetic acid (TCA) and 20 mM dithiothreitol (DTT). The pellets were washed with 20 mM DTT in 100% acetone, air-dried, and solubilized in 1 ml of rehydration buffer containing 7 M urea, 2 M thiourea, 4% CHAPS, 30 mM Tris, and 40 mM DTT. The protein concentration was measured using the 2D-quant kit (GE Healthcare, Chicago, Illinois, USA). The samples were stored at −20°C for further analysis.

#### Two-Dimensional Gel Electrophoresis

For the first dimension, the IPG strips (linear pH 3–10, 13 cm, GE Healthcare) were rehydrated in 250 μl of rehydration buffer containing 150 μg of protein sample and 1% ampholyte with pH 3–10 (GE Healthcare). The isoelectric focusing (IEF) was performed using Etton IPGphor 3 (GE Healthcare). After IEF, the strips were subsequently equilibrated in 5 ml of equilibration buffer [50 mM Tris-HCl pH 8.8, 6M urea, 30% (v/v) glycerol, 2% (w/v) sodium dodecyl sulfate (SDS)] containing 1% (w/v) DTT for 15 min followed by 15 min incubation in 5 ml equilibration buffer with 2.5% (w/v) iodoacetamide. The second dimension was performed using Ettan™ Daltsix electrophoresis unit (GE Healthcare). Strips were placed on the gels and overlaid with 0.4% molten agarose. Protein separation was carried out using an electric current of 2 W per gel for 45 min, followed by 12 W per gel until the dye front reached the gel bottom. Gels were stained by colloidal Coomassie Brilliant Blue G-250.

#### Image Analysis

The gels were scanned with an Amersham imager, and the images were analyzed using the ImageMaster 2D Platinum v7 software (GE). ANOVA was carried out at a threshold of *p* ≤ 0.05, power ≥0.8, and ≥1.5-fold change in average spot volume between control and heat treatments for further MS analysis.

#### In-gel Digestion and Protein Identification

According to Wang et al. (2015), the differentially expressed protein spots between control and heat stress were subjected to the in-gel trypsin digestion. The digested spots were loaded on the MALDI plate, and the samples were analyzed using the AB SCIEX TOF/TOF 5800 (SCIEX) mass spectrometer using an accelerating voltage of 25 kV and 200 spectra/s speed for the peptide mass fingerprinting. Laser power was modulated to have the best signal-to-noise ratio. The Protein Pilot software (SCIEX) was used to extract and process the peptide mass peaks from the spectrum. Protein identification was performed by searching the SWISSPROT database using an in-house Mascot server (http://www.matrixscience.com) integrated with MaxQuant software v1.5.3.8. Carbamidomethyl cysteine was chosen for global modification; oxidation of methionine was chosen for variable modification; one missed cleavage was allowed; and minimum peptide length was set to seven peptides.

### Statistical Analysis

Statistical significance among all the biochemical assays was performed by using one-way ANOVA followed by Tukey's test using SPSS25.0. However, all graphical and statistical significance representations were performed by MS-Excel 2019, showed as mean ± SEM.

## Results

### Effect of Heat Stress on Antioxidants and Related Metabolites

**Figure 1:**
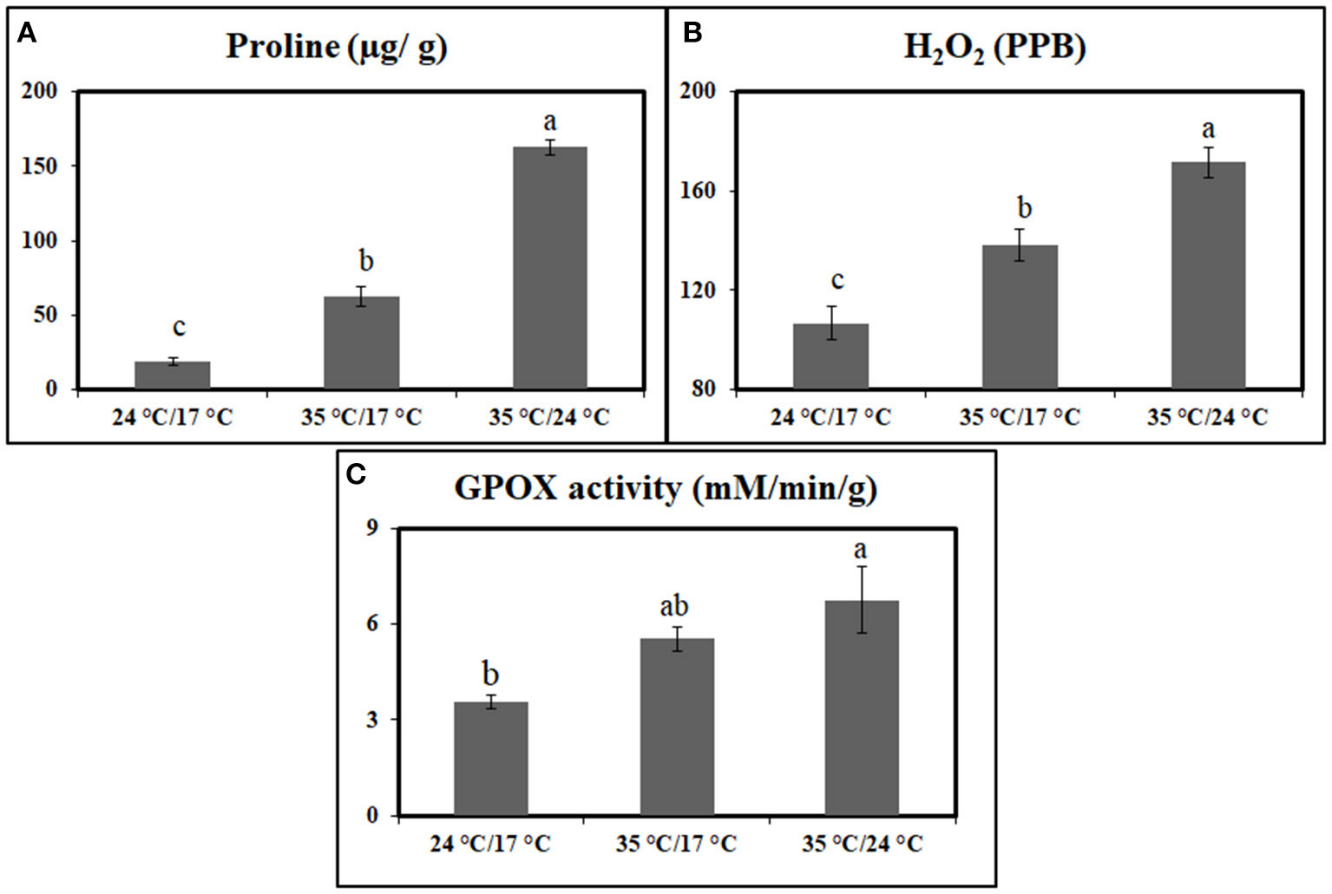
Graph representing the effect of heat stress during the grain-filling period on antioxidants and antioxidant enzymes between control, day heat stress, and day–night combined heat stress. **(A)** Proline (μg/g); **(B)** H_2_O_2_ (ppb). **(C)** Glutathione peroxidase (mM/min/g). Values were given in Mean ± SE. Variables with the same letter are not significantly different from each other at *P* ≤ 0.05.

The antioxidant measurements in the flag leaves indicated that proline and H_2_O_2_ increased significantly under heat stress when compared with control plants at 7 DAA. Under day stress, proline and H_2_O_2_ content were increased by 238 and 29%, respectively (Figures 1A,B). The increase in concentration was higher under day–night stress, i.e., 780% in case of proline and 61% for H_2_O_2_ (Figures 1A,B).

GPOX activity also showed a significant increase under heat stress when compared with control. Under day stress and day–night stress, GPOX activity showed 1.5- and 1.8-fold increase, respectively, as compared with control (Figure 1C).

### Effect of Heat Stress on Grain-Filling Duration and Yield-Related Parameters

**Figure 2:**
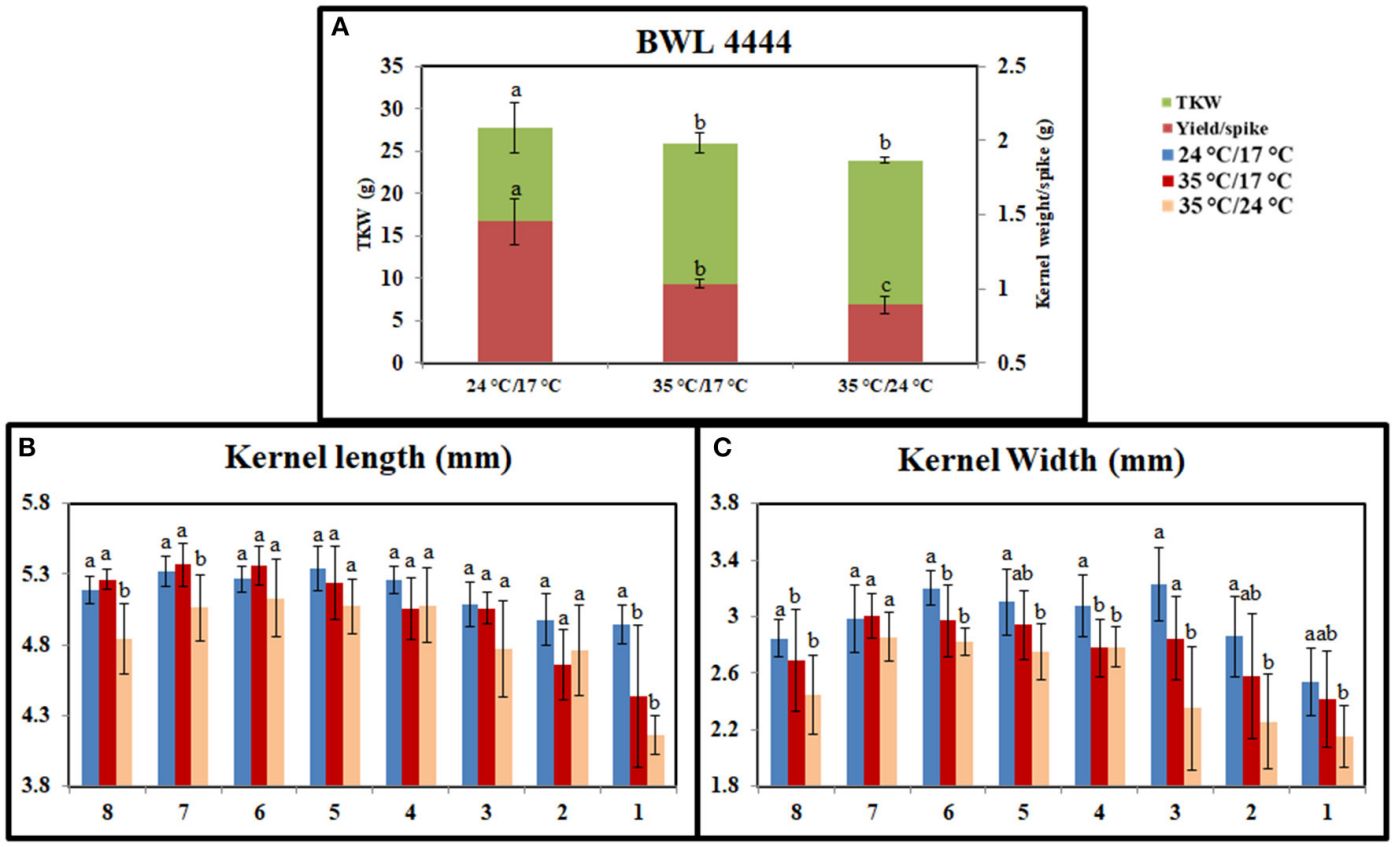
Graph representing the effect of heat stress during grain-filling period on yield parameters between control, day heat stress, and day–night combined heat stress. **(A)** Kernel weight/spike in grams (g) and thousand kernel weight (TKW) in grams (g). **(B)** Kernel length (mm). **(C)** Kernel width (mm). The x-axis in **(B,C)** indicates bottom spikelet (8) to top spikelet (1). Values were given in Mean ± SE. Variables with the same letter are not significantly different from each other at *P* ≤ 0.05.

Heat stress significantly affected the grain-filling period. Under the controlled conditions, plants took 35 days to complete the grain-filling period. It was reduced to 28 days under day stress and 25 days under day–night stress. The mean reductions in kernel weight per spike were 32 and 41% under day stress and day–night stress, respectively. Also, the reductions in the TKW were 11 and 18% under day stress and day–night stress, respectively (Figure 2A). Heat stress affected the grain seed length and width as well. Under day stress, the reductions in seed length and width from the top spikelets were higher than middle spikelets compared with control (Figures 2B,C). Under day–night stress, the seed length and the width from both top and bottom spikelets observed higher reduction than middle spikelets as compared with control.

### Effect of Heat Stress on Gene Expression

**Figure 3:**
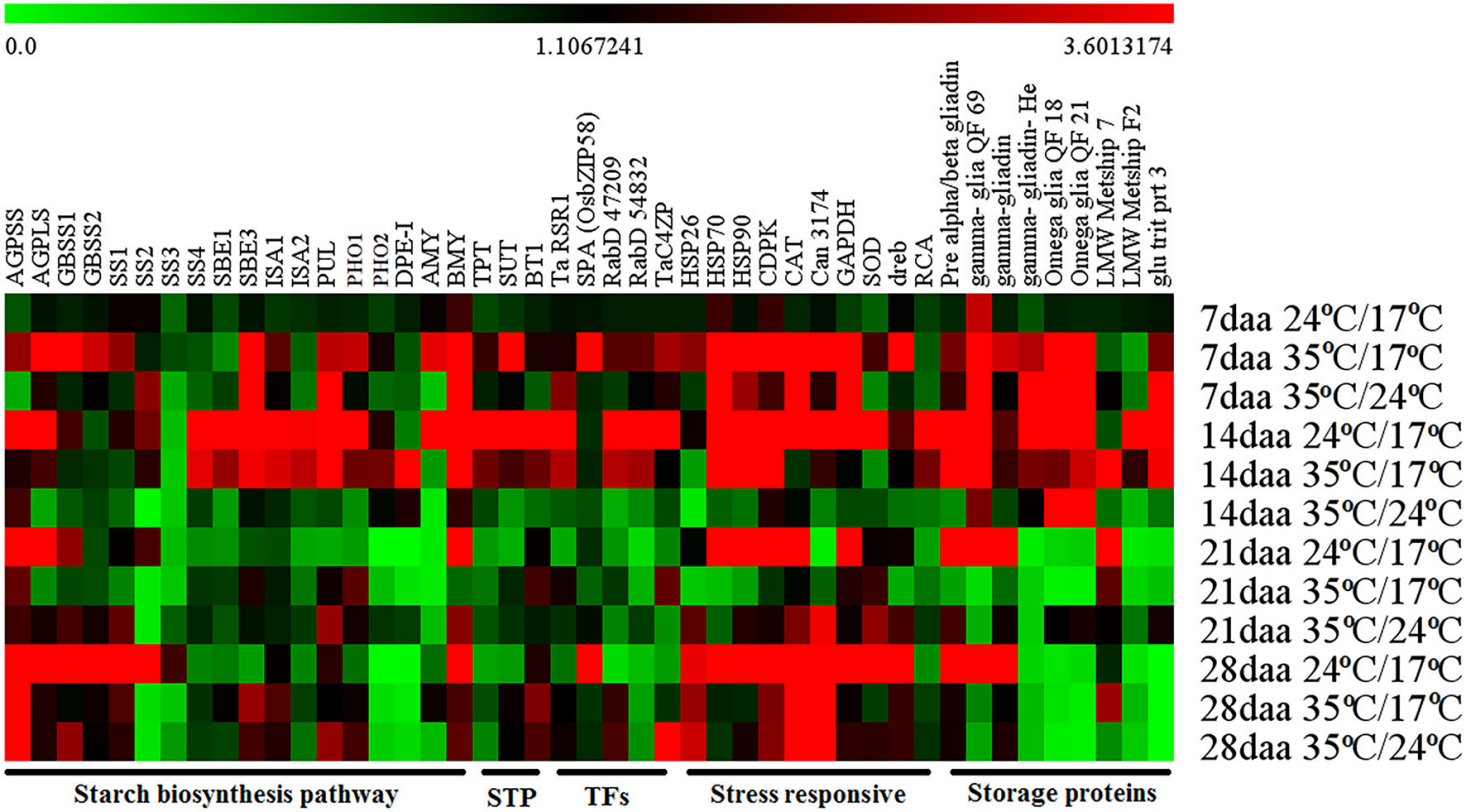
Heat map obtained from RT-qPCR representing the gene expression at different grain developmental stages from various temperature regimes.

The expression profiles of different pathway genes from four developmental stages and three temperature regimes were monitored by RT-qPCR (Figure 3; Supplementary Table 3).

#### Starch Biosynthesis Pathway Genes

The genes involved in the starch biosynthetic pathway were divided into four categories based on the expression pattern in the control group. The first category included genes with high expression at the early grain developmental stage, i.e., 14 DAA and relatively less expression in later stages, i.e., 21 DAA and 28 DAA. It included soluble starch synthase 4 (*SS4*), starch branching enzymes (*SBE1* and *SBE3*), isoamylase (*ISA1* and *ISA2*), pullulanase (*PUL*), starch phosphorylase (*PHO1*), and α-amylase (*AMY*). Under day stress, the expression of first category genes was higher at 14 DAA, with few genes started expressing at 7 DAA (*SBE3, ISA1, PUL*, and *PHO1*). The expression of these genes was reduced at later developmental stages with few showing minimal expression (*SBE3, PUL*, and *PHO1*) at 21 DAA and others (*SBE1, SBE3, ISA, PUL*, and *PHO1*) at 28 DAA. Under day–night stress, *SBE3, PUL*, and *BMY* showed relatively high expression at the early stage, i.e., 7 DAA, whereas the expression of other genes was low throughout the grain developmental stages (Figure 3).

The second category included genes with high expression toward the later stage of seed development (28 DAA) with minimal expression during early stages, i.e., 7 DAA, 14 DAA, and 21 DAA. They were granule-bound starch synthase (*GBSSI* and *GBSSII*) and soluble starch synthase (*SS1, SS2*, and *SS3*). Under day stress, the expression of *GBSSI, GBSSII*, and *SS1* genes was observed at 7 DAA with a limited expression at 28 DAA. The expression of *SS2* was observed only at 7 DAA and 14 DAA, and relatively lower expression of *SS3* was observed at 7 DAA. Under day–night stress, very low expression of *GBSSI, GBSSII*, and *SS1* was observed at 7 DAA and 14 DAA, but the relatively higher expression was observed at 21 DAA and 28 DAA. The expression of *SS2* under day–night stress was only observed at 7 DAA, and the expression of *SS3* was not observed throughout the development (Figure 3).

The third category included genes with high expression throughout the grain development, i.e., 14 to 28 DAA. It included ADP glucose pyrophosphorylase (*AGP*) large subunit (*AGPL1*), small subunit (*AGPS1*), and β-amylase (*BMY*). Under day stress, the expression of *AGPS1* was high at 7 DAA and 28 DAA and low at 14 DAA and 21 DAA, while the expression of *AGPL1* was high at 7 DAA and reduced after that with a limited expression at 28 DAA. The expression of *BMY* was observed at 7 DAA and 14 DAA, and subsequently, its expression was reduced. Under day–night stress, *AGPS1* and *AGPL1* had less expression from 7 to 21 DAA. At 28 DAA, they had a relatively higher expression. The expression of *BMY* was observed to be high at 7 DAA and low at 14–28 DAA (Figure 3).

The fourth category included genes with low to negligible expression throughout the development, i.e., starch phosphorylase 2 (*PHO2*) and disproportionating enzyme (*DPE-1*). Under day stress, these genes showed limited expression at 7 DAA, relatively higher expression at 14 DAA, and negligible expression at 21 and 28 DAA. Under day–night stress, these genes had limited expression at 14 and 21 DAA and negligible expression at 7 and 28 DAA (Figure 3).

#### Starch Transporter Genes

Under control conditions, the expression of starch transporter genes, i.e., triose phosphate translocator (*TPT*), sucrose transporter (*SUT*), and brittle-1 transporter (*BT-1*), was high at 14 DAA. Under day stress, these genes showed high expression during 7 and 14 DAA and minimal expression at 28 DAA, but at 14 DAA their expression was relatively lower than control. Under day–night stress, these genes had low to negligible expression throughout the development (Figure 3).

#### Stress-Responsive Genes

Under control conditions, most of the stress response genes, i.e., *HSP* (*HSP26, HSP70*, and *HSP90*), calcium-dependent protein kinase–signaling molecule (*CDPK*), catalase (*CAT*), hypothetical protein candidate (*Can 3174*), glyceraldehyde-3-phosphate dehydrogenase (*GAPDH*), superoxide dismutase (*SOD*), and dehydration-responsive element–binding proteins (*dreb*) had a high expression from 14 DAA to 28 DAA except for *Can3174* that had a negligible expression at 21 DAA. Under day stress, the peak expression of these genes was observed at 7 DAA. *HSP70, HSP90*, and *CDPK* showed high expression at 14 DAA, and *CDPK, CAT*, and *Can3174* showed high expression at 28 DAA. Under day–night stress, the expression of *HSP70, HSP90, CDPK, CAT, Can3174*, and *GAPDH* was high at 7 DAA. The expression of *HSP26, CAT, Can-3174*, and *SOD* was high at 21 DAA and 28 DAA. The expression of rubisco activase (*RCA*) was highest at 14 DAA under control and day stress conditions, but it had reduced expression during other stages. Under day–night stress, the expression of *RCA* was very less throughout the grain development (Figure 3).

#### Storage Proteins

Under control conditions, the γ-gliadin QF 69 had shown its expression throughout the development, but under day and day–night stress its expression was limited to 7 and 14 DAA. Pre-α/β gliadin and γ-gliadin had high expression during 14 and 21 DAA under control conditions. However, under day stress, the expression of these genes was observed at 7 and 14 DAA with a limited expression at 28 DAA. Under day–night stress, the expression of pre-α/β gliadin and γ-gliadin was observed at 7, 21, and 28 DAA. The expression levels of γ-gliadin He, ω-gliadin QF-18, ω-gliadin QF21, and glu trit prt 3 were high at 14 DAA under control conditions, but under day stress their expression was high during 7 and 14 DAA. At day–night stress, their expression was high at 7 DAA, and only γ-gliadin He, ω-gliadin QF-18, and ω-gliadin QF21 showed expression at 14 DAA. Under control conditions, LMW metship 7 had a high expression at 21 DAA, but under day stress it had high expression at 14–28 DAA. Under day–night stress, the expression of LMW metship 7 was low throughout the developmental stages with relatively high expression at 21 DAA. The expression of LMW metship F2 was high at 14 DAA under control and day stress conditions, but its expression was low in day–night stress throughout the development (Figure 3).

#### Transcription Factors

The expression of rice starch regulator (*TaRSR1*), Rab GTPases (RabD54832 and RabD47209), and *TaC4ZFP* (C4-type zinc finger transcription factor) was high at 14 DAA under control conditions. Under day stress, these genes had high expression during 7 and 14 DAA; however, at 14 DAA, their expression was relatively lower than that compared with control. Under day–night stress, these genes had minimal to low expression throughout the development except for *TaC4ZFP*, which showed high expression at 28 DAA. The expression of basic leucine zipper SPA (*Os bZIP 58*) was high at 28 DAA under control condition, but under day stress its expression was shifted to 7 DAA. The expression of *Os bZIP 58* was very low throughout the grain development under day–night stress (Figure 3).

### Protein Content and Profiles

The concentration of albumins–globulins and gliadins increased by 42% and 51% (under day stress) and 16 and 21% (under day–night stress), respectively. But the concentration of glutenins decreased by 7 and 29% under day stress and day–night stress, respectively. Overall, the total protein content increased under day stress; however, it remained unchanged under day–night stress as compared with control (Supplementary Table 4).

**Figure 4:**
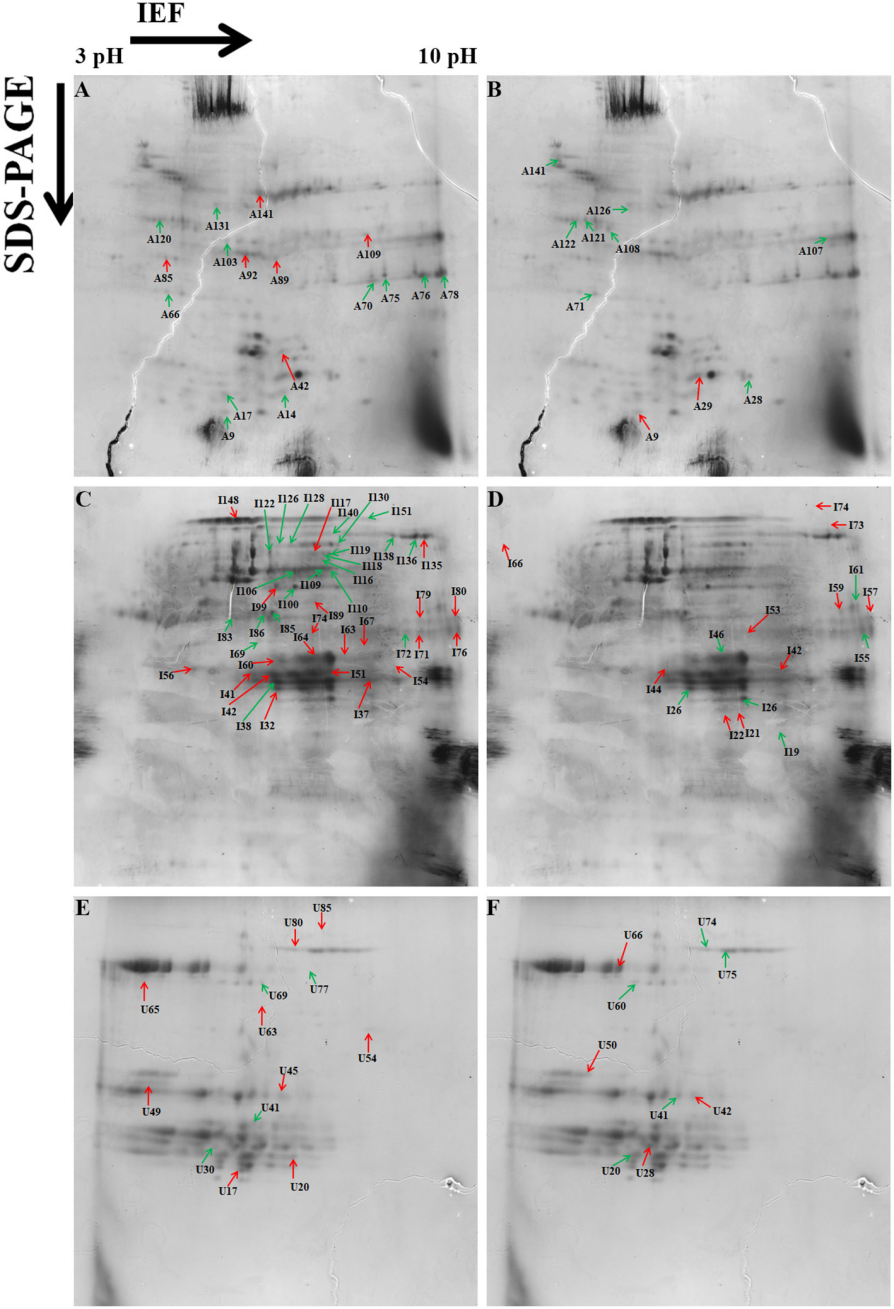
Representative two-dimensional electrophoresis (2-DE) gels of the wheat grain proteins analyzed by Protein Pilot software from different protein fractions. **(A)** Albumins–globulins day stress, **(B)** gliadins day stress, **(C)** glutenins day stress, **(D)** albumins–globulins day–night stress, **(E)** gliadins day–night stress, and **(F)** glutenins day–night stress. Red-colored arrows indicate upregulated protein spots between control and treatment. Green-colored arrows indicate downregulated protein spots between control and treatment.

**Figure 5:**
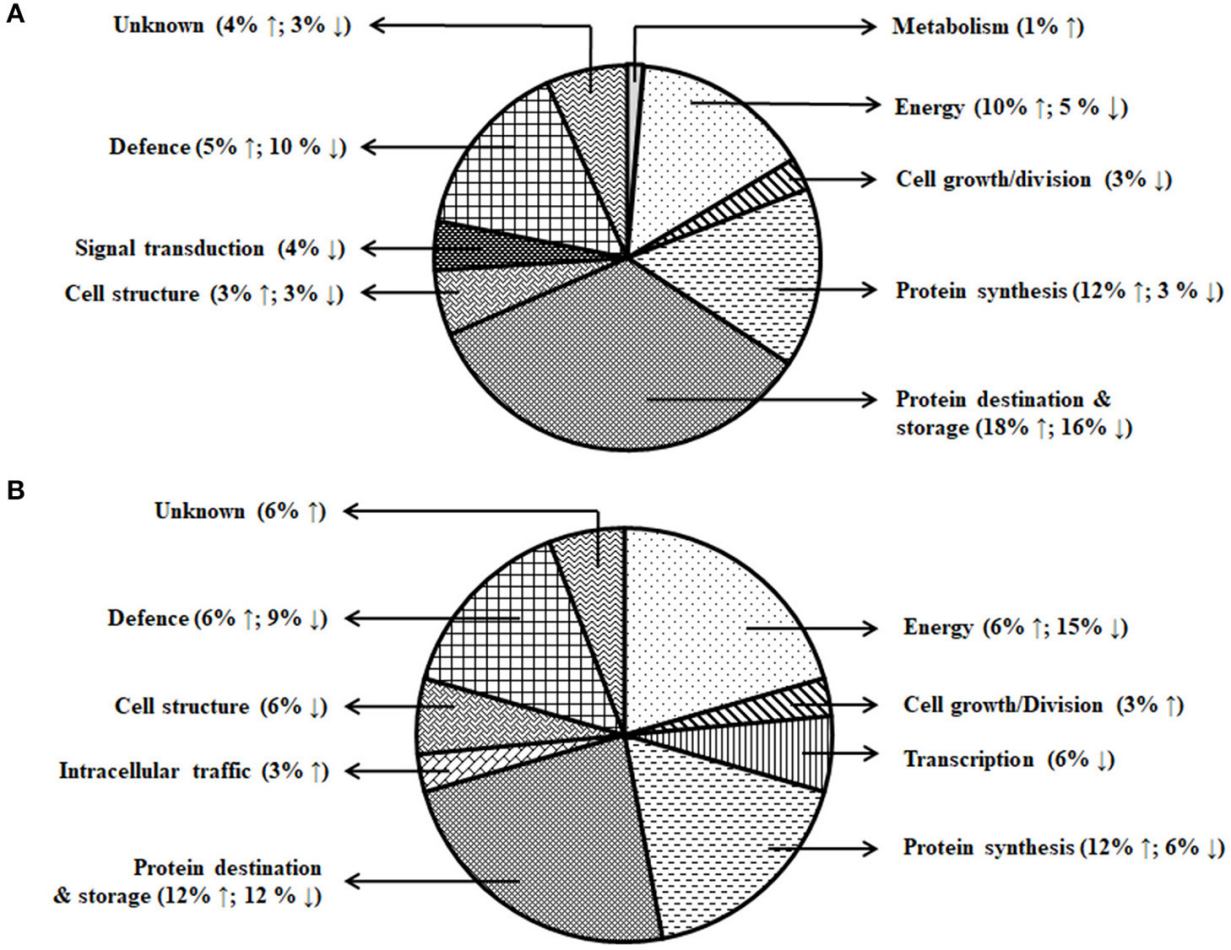
Functional classification of the differentially expressed proteins. **(A)** Day stress and **(B)** day–night stress. Proteins are classified according to Bevan et al. (1998).

A total of 153 differentially expressed protein spots with a fold change >1.5 were detected under day stress compared with control. Out of 153, 32 spots (13 up- and 19 downregulated) in albumin–globulin fraction, 77 spots (36 up- and 41 downregulated) in gliadins, and 44 spots (29 up- and 15 downregulated) in glutenins were detected (Figures 4A–C; Table 1). Only 73 out of 153 spots (39 up- and 34 downregulated) were identified by mass spectrometry (MS). Under day–night stress, 95 spots were detected to have a fold change of >1.5. About 28 spots (3 up- and 25 downregulated) in albumin–globulin fraction, 38 spots (16 up- and 22 downregulated) in gliadins, and 29 spots (18 up- and 11 downregulated) in glutenins were observed (Figures 4D–F; Table 2). Out of 95 spots, only 32 (15 up- and 17 downregulated) were identified by MS. According to Bevan et al. (1998), these protein spots were classified into different groups: metabolism, energy, cell growth/division, transcription, protein synthesis, protein destination and storage, intracellular traffic, cell structure, signal transduction, defense, and unknown functions (Figure 5).

**Table 1:** List of differentially expressed proteins in mature grains under day heat stress (35 /17°C vs. 24/17°C) as analyzed by matrix-assisted laser desorption/ionization time-of-flight mass spectroscopy.

**Spot #**	**Uniprot ID**	**Protein name**	**Fold change**	**Taxonomy**	**Cluster analysis**
I119	Q7XDH8	Cyclin-dependent kinase inhibitor 4	2.98↓	*Oriza sativa*	Cell growth/division
I122	Q402E2	Histone H3.3a	4.4↓	*Lilium longiflorum*	Cell growth/division
I138	P80798	65 kDa cell wall protein (fragment)	5.24↓	*Solanum lycopersicum*	Cell structure
I140	P82436	65 kDa cell wall protein (fragment)	2.2↓	*Nicotiana tobacum*	Cell structure
I60	P80798	65 kDa cell wall protein (fragment)	11.49↑	*Solanum lycopersicum*	Cell structure
U70	P80816	66 kDa cell wall protein (fragment)	3.03↑	*Solanum lycopersicum*	Cell structure
A103	P09407	Trypsin inhibitor MCI-3	4.04↓	*Momorica charantia*	Defense
A120	P93692	Serpin Z2B	1.58↓	*Triticum aestivum*	Defense
A14	P17314	Alpha-amylase/trypsin inhibitor CM3	2.81↓	*Triticum aestivum*	Defense
A17	P17314	Alpha-amylase/trypsin inhibitor	1.96↑	*Triticum aestivum*	Defense
A42	P16347	Endogenous alpha-amylase/subtilisin inhibitor	1.57↑	*Triticum aestivum*	Defense
A70	Q9FRV0	Basic endochitinase C	1.6↓	*Secale cereale*	Defense
A75	Q8L5C6	Xylanase inhibitor protein 1	2.25↓	*Triticum aestivum*	Defense
A76	Q9FRV0	Basic endochitinase C	2.86↓	*Secale cereale*	Defense
A78	Q9FRV0	Basic endochitinase C	1.96↓	*Secale cereale*	Defense
A9	O64392	Wheatwin-1	2.19↑	*Triticum aestivum*	Defense
U17	P81572	Defensin D1 (fragment)	14.41↑	*Spinacia oleracea*	Defense
A131	P05336	Alcohol dehydrogense	1.79↓	*Hordeum vulgare*	Energy
A141	Q75I94	Beta-glucosidase	1.62↑	*Oryza sativa japonica*	Energy
A66	P34937	Triose phosphate isomerase cytosolic	2.2↓	*Hordeum vulgare*	Energy
A89	Q0J8A4	Glyceraldehyde-3-phosphate dehydrogenase	6.45↑	*Oryza sativa japonica*	Energy
I130	Q9T2L1	Chlorophyll a–b binding protein (fragment)	1.68↓	*Spinacia oleracea*	Energy
I63	P11383	Ribulose bisphosphate carboxylase	2.51↑	*Triticum aestivum*	Energy
I83	P11383	Ribulose bisphosphate carboxylase large chain	6.1↓	*Triticum aestivum*	Energy
U20	Q3V517	Cytochrome b6-f complex subunit 6	33.85↑	*Acrus calamus*	Energy
U45	Q85A91	Cytochrome b6-f complex subunit 8	1.55↑	*Anthoceros formosae*	Energy
U65	P84733	Putative cyt c oxidase subunit 11 PS17	10.50↑	*Pinus strobus*	Energy
U85	P11383	Ribulose bisphosphate carboxylase large chain	38.43↑	*Triticum aestivum*	Energy
A85	Q8W593	Probable lactoylglutathione lyase	1.49↑	*Arabidopsis thaliana*	Metabolism
I106	P08488	Glutenin, high-molecular-weight subunit 12	2.97↓	*Triticum aestivum*	Protein destination and storage
I109	P08488	Glutenin, high-molecular-weight subunit 12	2.98↓	*Triticum aestivum*	Protein destination and storage
I116	P08488	Glutenin, high-molecular-weight subunit 12	1.93↓	*Triticum aestivum*	Protein destination and storage
I117	P08488	Glutenin, high-molecular-weight subunit 12	4.64↑	*Triticum aestivum*	Protein destination and storage
I118	P08488	Glutenin, high-molecular-weight subunit 12	1.55↓	*Triticum aestivum*	Protein destination and storage
I126	P08488	Glutenin, high-molecular-weight subunit 12	1.99↓	*Triticum aestivum*	Protein destination and storage
I128	P08488	Glutenin, high-molecular-weight subunit 12	3.05↓	*Triticum aestivum*	Protein destination and storage
I136	P02861	Glutenin, high-molecular-weight subunit PC256 (fragment)	2.25↓	*Triticum aestivum*	Protein destination and storage
I148	P0848	Glutenin, high-molecular-weight subunit PW212	3.31↑	*Triticum aestivum*	Protein destination and storage
I151	P08489	Glutenin, high-molecular-weight subunit PW212	3.37↓	*Triticum aestivum*	Protein destination and storage
I32	P18573	Alpha/beta-gliadin MM1	3.48↑	*Triticum aestivum*	Protein destination and storage
I37	P18573	Alpha/beta-gliadin MM1	63.88↑	*Triticum aestivum*	Protein destination and storage
I38	P02863	Alpha/beta-gliadin	2.37↓	*Triticum aestivum*	Protein destination and storage
I41	P18573	Alpha/beta-gliadin MM1	3.12↑	*Triticum aestivum*	Protein destination and storage
I51	P18573	Alpha/beta-gliadin MM1	4.29↑	*Triticum aestivum*	Protein destination and storage
I54	P18573	Alpha/beta-gliadin MM1	8.98↑	*Triticum aestivum*	Protein destination and storage
I64	P04727	Alpha/beta-gliadin clone PW8142	1.61↑	*Triticum aestivum*	Protein destination and storage
I71	P04730	Gamma-gliadin (fragment)	4.62↑	*Triticum aestivum*	Protein destination and storage
I72	P16315	Glutenin, low-molecular-weight subunit PTDUCD1	4.76↓	*Triticum aestivum*	Protein destination and storage
I74	P04730	Gamma-gliadin (Fragment)	11.77↑	*Triticum aestivum*	Protein destination and storage
I76	P10385	Glutenin, low-molecular-weight subunit	1.77↑	*Triticum aestivum*	Protein destination and storage
I79	P10385	Glutenin, low-molecular-weight subunit	6.38↑	*Triticum aestivum*	Protein destination and storage
I80	P04729	Gamma-gliadin B-I	5.26↑	*Triticum aestivum*	Protein destination and storage
I86	P12615	12S seed storage globulin 1	2.34↓	*Avena sativa*	Protein destination and storage
U77	P02861	Glutenin, high-molecular-weight subunit PC256 (Fragment)	1.77↓	*Triticum aestivum*	Protein destination and storage
A109	Q0P3L5	50S ribosomal protein chloroplast L14	2.56↑	*Ostreococcus tauri*	Protein synthesis
A92	Q0P3L5	50S ribosomal protein L14	2.23↑	*Ostreococcus tauri*	Protein synthesis
I100	P82452	60S ribosomal protein L26 (fragment)	7.69↓	*Spinacia oleracea*	Protein synthesis
I135	Q6ENF2	50S ribosomal protein L33	1.5↑	*Oriza nivara*	Protein synthesis
I42	P82452	60S ribosomal protein L26 (fragment)	3.25↑	*Spinacia oleracea*	Protein synthesis
I67	P82452	60S ribosomal protein L26 (fragment)	3.29↑	*Spinacia oleracea*	Protein synthesis
I89	P82452	60S ribosomal protein L26	3.93↑	*Spinacia oleracea*	Protein synthesis
I99	A0A317	30S ribosomal protein S16	3.08↑	*Coffea arabica*	Protein synthesis
U63	Q09X35	30S ribosomal protein S16	3.62↑	*Morus indica*	Protein synthesis
U69	P82452	60S ribosomal protein L26 (fragment)	2.48↓	*Spinacia oleracea*	Protein synthesis
U80	P82452	60S ribosomal protein L26 (fragment)	1.80↑	*Spinacia oleracea*	Protein synthesis
I85	Q6ZDF3	bZIP transcription factor TRAB1	6.03↓	*Oryza sativa* subsp. *japonica*	Signal transduction
U30	O24369	Early nodulin-40	3.97↓	*Sesbania rostrata*	Signal transduction
U41	O24369	Early nodulin-40	2.03↓	*Sesbania rostrata*	Signal transduction
I110	P85912	Unknown protein 12 (fragment)	2.32↓	*Pseudotsuga menziesii*	Unknown
I56	P85912	Unknown protein 12 (fragment)	3.55↑	*Pseudotsuga menziesii*	Unknown
I69	P85912	Unknown protein 12 (Fragment)	2.59↓	*Pseudotsuga menziesii*	Unknown
U49	Q32617	Uncharacterized 3.8 kDa protein in ycf12-psaM intergenic region	21.57↑	*Marchantia polymorpha*	Unknown
U54	Q1PFS7	Uncharacterized protein At1g24060	3.05↑	*Arabidopsis thaliana*	Unknown

**Table 2:** List of differentially expressed proteins in mature grains under day-night combined heat stress (35/24°C vs. 24/17°C) as analyzed by matrix-assisted laser desorption/ionization time-of-flight mass spectroscopy.

**Spot #**	**Uniprot ID**	**Name**	**Fold change**	**Species**	**Cluster analysis**
I22	Q7XSB2	Putative zinc finger CCCH domain-containing protein 29	3.81↑	*Oryza sativa subsp. japonic*	Cell growth/division
I46	P80798	65 kDa cell wall protein (fragment)	1.58↓	*Solanum lycopersicum*	Cell structure
I55	P82436	65 kDa cell wall protein (fragment)	3.76↓	*Nicotiana tabacum*	Cell structure
A121	P93692	Serpin-Z2B	3.08↓	*Triticum aestivum*	Defense
A122	P93692	Serpin-Z2B	6.04↓	*Triticum aestivum*	Defense
A28	Q07502	Defensin-like protein	1.28↓	*Glycine max*	Defense
A9	O64392	Wheatwin-1	1.92↑	*Triticum aestivum*	Defense
U20	Q03196	Cysteine proteinase inhibitor (fragment)	13.5↑	*Solanum tuberosum*	Defense
A108	Q0J8A4	Glyceraldehyde-3-phosphate dehydrogenase	5.8↓	*Oryza sativa subsp. japonica*	Energy
A141	P16098	Beta-amylase	1.09↓	*Hordeum vulgare*	Energy
A71	P34937	Triosephosphate isomerase	13.17↓	*Hordeum vulgare*	Energy
I21	Q49CC1	Ribulose bisphosphate carboxylase large chain	4.05↑	*Cuscuta sandwichiana*	Energy
I26	A4QJC3	Ribulose bisphosphate carboxylase large chain	1.86↓	*Aethionema cordifolium*	Energy
I74	A8Y9H8	Ribulose bisphosphate carboxylase large chain	8.27↑	*Lolium perenne*	Energy
U41	B0YPN8	Ribulose bisphosphate carboxylase large chain	6.85↓	*Aneura mirabilis*	Energy
I66	Q9C5P7	Protein transport Sec1a	21.73↑	*Arabidopsis thaliana*	Intra cellular traffic
I57	P10385	Glutenin, low-molecular-weight subunit	10.18↑	*Triticum aestivum*	Protein destination and storage
I59	P10385	Glutenin, low-molecular-weight subunit	2.06↑	*Triticum aestivum*	Protein destination and storage
I61	P10385	Glutenin, low-molecular-weight subunit	9.18↓	*Triticum aestivum*	Protein destination and storage
U50	P10385	Glutenin, low-molecular-weight subunit	6.6↑	*Triticum aestivum*	Protein destination and storage
U60	P08488	Glutenin, high-molecular-weight subunit 12	2.52↓	*Triticum aestivum*	Protein destination and storage
U66	P02861	Glutenin, high-molecular-weight subunit PC256 (Fragment)	1.66↑	*Triticum aestivum*	Protein destination and storage
U74	P08489	Glutenin, high-molecular-weight subunit PW212	2.28↓	*Triticum aestivum*	Protein destination and storage
U75	P08489	Glutenin, high-molecular-weight subunit PW212	2.98↓	*Triticum aestivum*	Protein destination and storage
A107	Q9SGE9	Probable phenylalanyl-tRNA synthetase beta chain	4.37↓	*Arabidopsis thaliana*	Protein synthesis
I32	A2T35	30S ribosomal protein S12	3.33↓	*Angiopteris evecta*	Protein synthesis
I53	B1VKE2	50S ribosomal protein L33	1.79↑	*Cryptomeria japonica*	Protein synthesis
I73	B2LMJ1	30S ribosomal protein S14	8.27↑	*Guizotia abyssinica*	Protein synthesis
U42	Q06RB1	50S ribosomal protein L33	2.81↑	*Jasminum nudiflorum*	Protein synthesis
A126	Q10Q26	B3 domain-containing protein	2.76↓	*Oryza sativa subsp. japonica*	Transcription
I42	P85912	Unknown protein 12 (fragment)	4.75↑	*Pseudotsuga menziesii*	Unknown
U28	P85912	Unknown protein 12 (fragment)	3.68↑	*Pseudotsuga menziesii*	Unknown

The energy group was represented by 15 and 20% of protein spots in the day stress and day–night stress, respectively. In day stress, seven protein spots belonging to energy group showed upregulation and four protein spots showed downregulation. The upregulated protein spots belonged to ribulose bisphosphate carboxylase large chain (RCL) (spot no: I63, U85), GAPDH (spot no. A89), beta-glucosidase (spot no. A141), cytochrome b6-f complex (C b6-f C) subunit-6 (spot no. U20), C b6-f C subunit 8 (spot no. U45), and putative Cyt c oxidase subunit 11 PS17 (spot no. U65). The identified downregulated protein spots were triose phosphate isomerase (TPI) (spot no. A66), alcohol dehydrogenase (spot no. A131), RCL (spot no. I83), and chlorophyll a–b binding protein (spot no. I130). In day–night stress, two protein spots that belonged to RCL (spot no. I21 and I74) were upregulated, and five protein spots, i.e., triosephosphate isomerase (A71), GAPDH (A108), and RCL (I21, I74, and U41) were downregulated.

The cell growth/division group was represented by 3% protein spots in both day stress and day–night stress conditions. The two downregulated protein spots under day stress belonged to cyclin-dependent kinase inhibitor 4 (CDKI) (I119) and histone H3.3a (I122). In day–night stress, a protein spot putative zinc finger CCCH domain-containing protein 29 (I22) showed upregulation.

The protein synthesis group was represented by 15% and 18% protein spots in day stress and day–night stress, respectively. Under day stress, 11 protein spots showed differential expression. Two protein spots showed downregulation and belonged to 60S ribosomal protein L26 (I100 and U69). The remaining nine upregulated protein spots were 50S ribosomal protein L14 (A92 and A109), 60S ribosomal protein L26 (I42, I67, I89, and U80), 30S ribosomal protein S16 (I99 and U63), and 50S ribosomal protein L33 (I135). In day–night stress, two out of identified six spots were downregulated. These were probably phenylalanyl-tRNA synthetase beta chain (A107) and 30S ribosomal protein S12 (I32). The remaining four upregulated spots were 50S ribosomal protein L33 (I53 and U42), 30S ribosomal protein S7 (I66), and 30S ribosomal protein S14 (I73).

The protein destination and storage group were represented by 35 and 24% of the protein spots in day stress and day–night stress, respectively. In day stress, 14 protein spots out of 25 identified were upregulated and 12 were downregulated. The upregulated protein spots were glutenin—LMW subunit (I76 and I79), glutenin—HMW subunit 12 (I117), glutenin—HMW subunit PW212 (I148), alpha-/beta-gliadin MM1 (I32, I37, I41, I51, and I54), alpha-/beta-gliadin clone PW8142 (I64), gamma-gliadin (I71, I74, and I80), and gamma-gliadin B-I (I80). The downregulated protein spots were alpha-/beta-gliadin (I38), 12S seed storage globulin 1 (I86), glutenin—LMW subunit PTDUCD1 (I72), glutenin—HMW subunit 12 (I106, I109, I116, I118, I126, and I128), glutenin—HMW subunit PC256 (I136 and U77), and glutenin—HMW subunit PW212 (I151). In day–night stress, four protein spots out of eight showed downregulation, and they were glutenin—LMW subunit (I61), glutenin—HMW subunit 12 (U60), and glutenin—HMW subunit PW212 (U74 and U75). The remaining four upregulated protein spots were glutenin—LMW subunit (I57, I59, and U50) and glutenin—HMW subunit PC256 (fragment) (U66).

The cell structure group was represented by 5 and 6% of the protein spots in day stress and day–night stress, respectively. The differentially expressed proteins spots belonged to 65 kDa cell wall protein (I138, I140, I60, U70, I46, and I55).

The defense group was represented by 15% of the protein spots in day stress and day–night stress. In day stress, 4 protein spots out of 11 were upregulated, and they were wheatwin-1 (A9), defensin D1 (U17), endogenous alpha-amylase/subtilisin inhibitor (A42), and alpha-amylase/trypsin inhibitor (A17). The other seven downregulated protein spots were alpha-amylase/trypsin inhibitor CM3 (A14), trypsin inhibitor MCI-3 (A103), serpin Z2B (A120), basic endochitinase C (A70, A76, and A78), and xylanase inhibitor protein 1(A75). In day–night stress, two out of five upregulated protein spots were wheatwin-1(A9) and cysteine proteinase inhibitor (U20). The other downregulated protein spots were serpin-Z2B (A121 and A122) and defensin-like protein (A28).

Under day stress, one protein spot (1%), probably lactoyl glutathione lyase (spot no. A85), belonging to metabolism group showed upregulation and three protein spots (4%) belonging to signal transduction group, bZIP transcription factor TRAB1 (I85) and early nodulin-40 (U30 and U41), showed downregulation. Under day–night stress, one protein spot (3%), protein transport Sec1a (I66), belonging to the intracellular transport group showed upregulation. The transcription group was represented by 6% of the protein spots under day–night stress. The two downregulated protein spots were B3 domain-containing protein (A126) and pentatricopeptide repeat-containing protein (I32).

The differentially expressed proteins detected under both day and day–night heat stress included proteins belonging to starch biosynthesis, cell wall synthesis, defense, and storage proteins. Cell wall and starch synthesis proteins were downregulated under both types of stresses; these included 65 kDA cell wall protein, TPI, RCL, and GAPDH. While protein synthesis-related proteins, i.e., 50s ribosomal proteins were mainly upregulated under both types of stress. Contrasting protein expression was observed for storage proteins and defense-related proteins as wheatwin-1, LMW glutenins, and gliadins were upregulated, and serpin Z2B and HMW-GS were downregulated in both the conditions (Table 3).

**Table 3:** List of differentially expressed proteins under both day and day–night heat stresses.

	**Downregulated proteins**	**Upregulated protein**
Cell structure	65 kDa cell wall protein	
Defense	Serpin Z2B	Wheatwin-1
Energy	Triose phosphate isomerase	
	RuBisCO large chain	
	Glyceraldehyde-3-phosphate dehydrogenase	
Protein storage and destination	HMW-GS	LMW-GS
		Alpha/beta-gliadin
		Gamma-Gliadin
Protein synthesis		50S ribosomal protein

## Discussion

Wheat grain-filling period is the most susceptible phase for high-temperature stress (Farooq et al., 2011). Previous agronomic trait-based studies had observed that heat stress retards seed growth during grain-filling period by affecting the biochemical events such as the ability of the grains to accumulate water (Kino et al., 2020) causing losses in grain yield and number (Prasad and Djanaguiraman, 2014; Bheemanahalli et al., 2019). Most of the studies had only provided periodic heat stresses (Chauhan et al., 2011; Yang et al., 2011; Liu et al., 2015; Rangan et al., 2020; Tomás et al., 2020) during the reproductive stages for studying their effect on morphological and physiological parameters, but the molecular mechanism behind the effect of heat stress on grain-filling period is less understood (Liu et al., 2015; Kino et al., 2020; Rangan et al., 2020). Only few studies are available that have used heat stress throughout the grain-filling period and studied either protein or gene profiles (Zhang et al., 2017). In this study, the effect of postanthesis day heat stress and day–night combined heat stress was observed throughout the grain-filling period, and the molecular mechanism involved in the plant heat stress response through both gene and protein profiles have been attempted for better understanding.

### Effect of Heat Stress on Metabolism and Yield

Reduction in yield is associated with lower photosynthesis and reduced starch accumulation (Wang et al., 2012). In the present study, the protein profiles showed the downregulation of RCL subunits and Cyt b6-f complex under day stress conditions, indicating reduced carbon fixation and photosynthesis. The downregulation of Cyt b6-f protein under heat stress condition had also been observed earlier in the case of wheat seedling (Luo et al., 2018). Similarly, the authors have observed that heat stress affected the expression of various starch biosynthesis pathway–related genes. The limited expression of *AGPL1, AGPS1, GBSSI, GBSSII, SS (1, 2*, and *3)*, and *ISA* under heat stress conditions caused a reduction in the synthesis of ADP-glucose and conversion to amylose and amylopectin that led to a reduced starch accumulation. Heat stress-associated reduction in the expression of genes involved in the starch biosynthetic pathway such as *AGPL, AGPS* (Impa et al., 2019), and *SS* (Hurkman et al., 2003) has also been reported earlier. Similarly, the expression of starch transporter genes (*SUT, BT-1*, and *TPT*) under heat stress was initiated earlier at 7 DAA under day stress conditions in contrast to the control condition where their expression was observed at 14 DAA, indicating that the starch synthesis occurred during early developmental stages. However, under the day–night stress, the expression of these genes was minimum throughout the grain development, indicating the reduced transportation of starch precursors that has led to decreased starch levels and grain yield. Reduction in expression levels of *SUS* and *SUT* genes was also observed in rice under heat stress leading to reduced kernel weight (Zhang C. X. et al., 2018). The present study also observed the limited expression of BMY, an enzyme that converts starch to glucose under heat stress conditions, indicating reduced supplementation of energy for metabolic pathways. The present study also observed lower expression of the transcription factors (*TaRSR1* and *OsbZIP58*) that regulate the starch biosynthesis (Kang et al., 2013; Wang et al., 2013; Singh et al., 2015). It can be postulated that defense response activated by heat stress has reduced the expression of the starch synthesis related to the regulatory transcription factors. This has reduced the expression of biosynthetic, processing, and transport associated genes, ultimately leading to the lower starch deposition.

The protein profiles have also indicated upregulation of *GAPDH*, a rate-limiting enzyme in the glycolysis pathway under day stress, and downregulation under day–night stress indicating that its activity increases under day stress but reduces on higher stress. Previous studies had also observed an increase in GAPDH levels under heat stress in rice seedling (Han et al., 2009) and wheat seedling (Wang et al., 2015). TPI, another important glycolytic pathway enzyme that catalyzes the reversible interconversion of dihyroxyacetone phosphate to glyceraldehyde 3-phosphate, was downregulated in both day and day–night stresses. The decreased abundance of GAPDH and TPI in glycolysis leads to the oxidation of glucose-6-phosphate, which further enters the pentose phosphate pathway for the generation of NADPH. Studies also reported the decreased abundance of TPI under heat stress conditions (Yang et al., 2011). It has been reported that several glycolytic enzymes including TPI were increased at 10 DAA under heat stress condition (Hurkman et al., 2009). This is in contrast to the present results, which might be due to the duration of stress exposure or being cultivar-specific.

Increase in grain protein content had been observed under heat stress (Ashraf, 2014). From our data, we observed that the genes related to gliadins (α/β, γ-gliadins) showed high expression during early developmental stages, i.e., 7 and 14 DAA under heat stress conditions, but under control conditions, their expression was observed throughout the grain development. ω-gliadin showed high expression only at 14 DAA under control conditions, but its expression under stress conditions was observed at 7 and 14 DAA (day alone) and 7, 14, and 21 DAA (day–night stress) that indicated higher expression of ω-gliadins under stress conditions. LMW metship 7, a precursor gene for LMW glutenins, had a high expression at 21 DAA under control conditions, but its expression was observed at 14, 21, and 28 DAA under day stress conditions that indicated higher LMW-GS synthesis under stress conditions. Glu trip prt 3, a precursor for HMW glutenins (Zeltner et al., 2009), had high expression at 14 DAA under control conditions. An increased expression was observed at 7 DAA under heat stress with very low expression in later stages (Figure 3). Overall, HMW-GS proteins were downregulated, and LMW-GS, α/β, and γ-gliadin proteins were found to be upregulated under both stress conditions. Previous studies had also observed continuous synthesis of both gliadin and glutenins throughout the grain-filling period under heat stress (Bernardin et al., 1995). The study of Altenbach et al. (2002) indicated that under high-temperature regimens, transcripts from all seed storage protein gene families, appeared slightly earlier, but the timeframe of accumulation was shorter. Majoul et al. (2003) also observed decreased synthesis of glutenins and stable or increased synthesis of gliadins under heat stress. This might be due to the earlier synthesis of gliadins and LMW-GS compared with HMW-GS during grain development (Mazzeo et al., 2017). Heat stress reduces grain-filling period and advances the expression of biosynthetic genes, and thus gliadins and LMW-GS might be less affected or have increased as compared with HMW-GS. Our results also observed a reduction in glutenin-to-gliadin ratio of the matured grain under heat stress by 35–40% (Supplementary Table 4). Previous studies had reported a reduction in glutenin/gliadin ratio (Blumenthal et al., 1993) and HMW/LMW ratio (Don et al., 2005) under heat stress. Decreased glutenin/gliadin and HMW/LMW ratio reduces the baking quality (Balla et al., 2011; Garg et al., 2014).

Our protein profiles also found a higher abundance of ribosome-binding proteins that belong to the protein synthesis group under both heat stress conditions. Similar observations for ribosome-binding proteins had also been reported earlier in the matured grains after 2-h stress (Zhang Y. et al., 2018).

**Figure 6:**
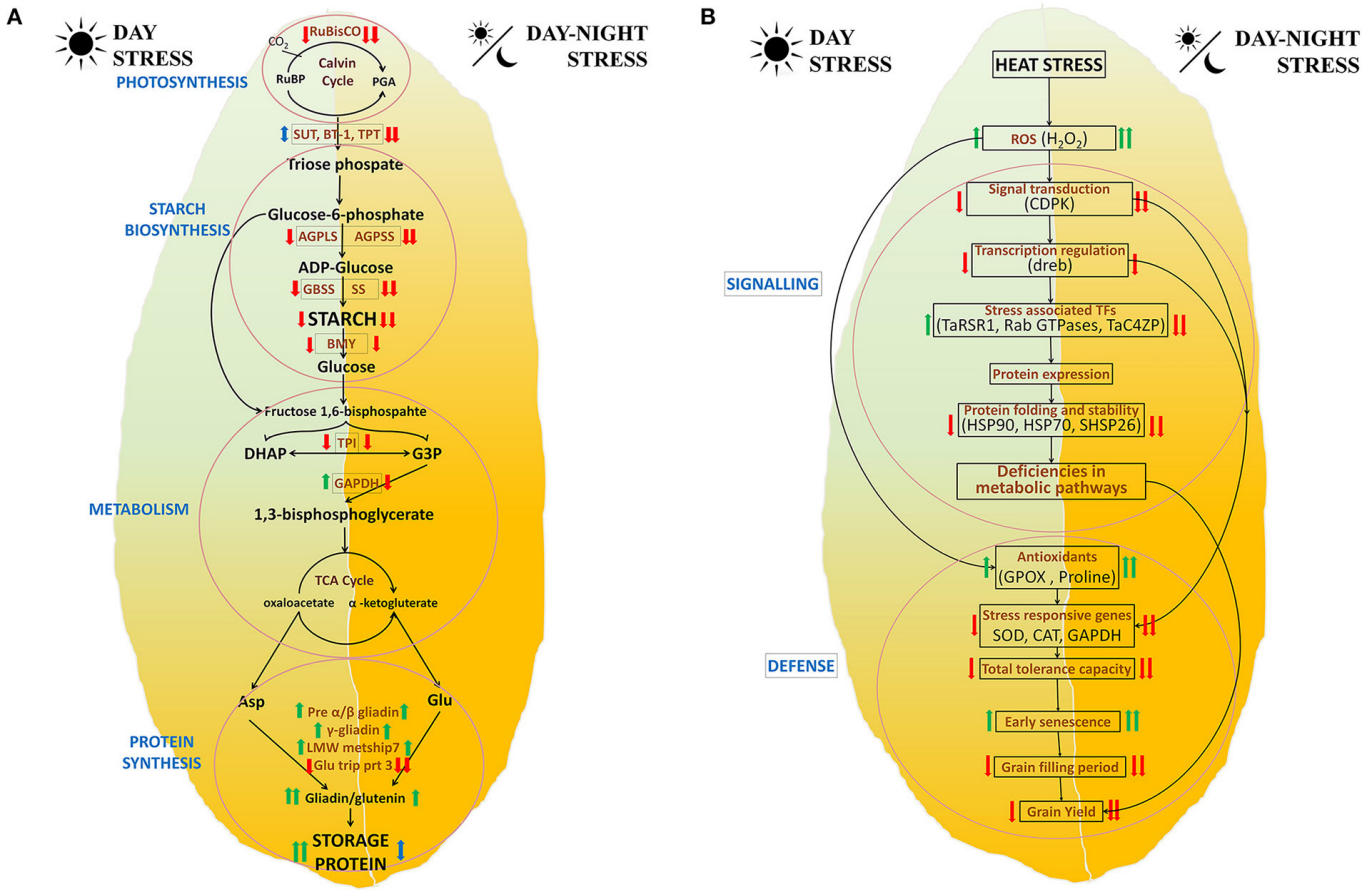
Proposed mechanism of the postanthesis day and day–night heat stress responses in BWL4444. **(A)** Biosynthesis pathway and **(B)** stress response.

The effect of heat stress on various metabolic pathways has been graphically represented in Figure 6A. It indicates that a reduction in photosynthesis affects starch and protein biosynthesis. Combined stress induces a higher effect as compared with a single stress. It involves the interplay of several genes involved in multiple pathways including photosynthesis, transport of photosynthates to cellular organelles including amyloplasts, starch biosynthesis, glycolysis, protein precursors, and protein synthesis.

### Effect of Heat Stress on the Stress Response

Plants have adapted to various mechanisms to handle abiotic stresses at the morphological, physiological, biochemical, and genetic levels, for allowing them to survive under adverse conditions (Huber and Bauerle, 2016). In the present study, the increase in H_2_O_2_ concentration under stress conditions increased reactive oxygen species, which further triggers several signaling cascades (Lamaoui et al., 2018). Increased H_2_O_2_ content led to the activation of GPOX and an increase in proline content. It is a defense mechanism by plants that reduces the H_2_O_2_ concentration inside the cell. Our gene expression data showed high expression of *CDPK* until 14 DAA under day stress and 7 DAA under day–night stress in contrast to control where its expression was observed throughout the development. *CDPK* is a stress response gene that modulates the expression of several genes and proteins throughout plant growth and development (Asano et al., 2012). The reduced duration of *CDPK* expression indicated that under prolonged heat stress, the plant lost its ability to trigger other genes for the proper functioning of the cell. The increase in expression of *CDPK* after 2 h heat stress had also been reported earlier (Kumar et al., 2014). Similarly, the limited expression of transcription factor *TaC4ZFP* under heat stress indicated the loss of abiotic stress response as *TaC4ZFP* is the major heat stress response gene known to be a regulator for ABA-responsive signaling under heat stress (Li et al., 2013). The previous study observed an upregulation of 60-fold after 1.5 h heat stress, but its expression reduced after 5 h of treatment. The present study has also observed very low expression of other regulatory genes related to protein trafficking throughout the seed development (Rab GTPases), indicating their improper regulation under stress conditions. Tyler et al. (2015) also showed decreased expression of Rab GTPases under heat stress, thus stopping the regulation of vesicles carrying seed storage proteins from endoplasmic reticulum to Golgi complex. To sum up, heat stress has enhanced various stress-responsive genes and proteins but cannot maintain their expression under prolonged stress duration.

Our gene expression data have also shown limited expression of *HSP90* and *HSP70* under heat stress conditions (until 14 DAA), indicating reduced processing and folding of proteins during late developmental stages. Kumar et al. (2014) had observed an increase in expression of *HSP70* and *HSP90* after 2 h of heat stress post anthesis. Chloroplast-specific *sHSP26*, which helps in protecting the PS-II under heat stress (Heckathorn et al., 2002), had also shown reduced expression under continuous day and day–night stresses in later developmental stages indicating damage to the PS-II and reduction in the photosynthesis. *RCA*, a key chaperon that helps in the proper functioning of *RUBISCO* enzyme (Perdomo et al., 2017), was also downregulated under both day and day–night stress, which indicates decreased photosynthetic capacity (Supplementary Table 3). Kumar R. et al. (2016); Kumar R. R. et al. (2016) had observed an increase in *RCA* expression under 2 h of high-temperature stress; however, in the present study, the expression was observed throughout the grain development. The other heat response genes such as *CAT, Can3174, SOD*, and *GAPDH*, which are helpful in signaling and reduction of reactive oxygen species produced due to heat stress, had also shown increased expression during early developmental stages but were unable to maintain throughout the grain development. Overall, the continuous exposure to heat stress reduced the expression of proteins involved in folding and processing and increased the ROS, thereby decreasing the photosynthesis and metabolic processes.

The protein profiles also indicated downregulation of tumor suppressor protein, CDKI, under heat stress indicating enhanced cell division. Kamal et al. (2010) had observed enhanced expression of CDKI under abiotic stress. While reports on the reduction of the expression of cyclins by enhancing the inhibitory phosphorylation of CDKs are also available (Rowley et al., 1993), histone H3 3a also got downregulated under heat stress, which indicates the instability of DNA structure and defects in transcription regulation. The downregulation of histone H3 3a leads to irregularities in histone acetylation and methylation, which further affects the transcription regulation (Kong et al., 2020). Proteins with antifungal activity like wheatwin-1 (Caporale et al., 2004) and defensin D1 had shown upregulation under day stress. However, defensin D1 showed downregulation under day–night stress. Previous studies also observed an increase in transcript levels of wheatwin under heat stress (Altenbach et al., 2007). However, basic endochitinase c and xyalanase inhibitors with antifungal properties (Ohnuma et al., 2002) showed downregulation. Alpha-amylase inhibitor was highly expressed under heat stress. Studies had also reported an increased abundance of alpha-amylase inhibitors under heat stress (Laino et al., 2010). Serpins that have inhibitor function for chymotrypsin and trypsin may also act as allergens. Our studies have shown downregulation of serpins under heat stress. Laino et al. (2010) had observed upregulation of serpins under heat stress. The proteins related to the cell wall (65 kDa cell wall protein), which help in binding to tubulins, had shown downregulation under heat stress which indicates the loss of cell structure.

The effect of heat stress on various stress-related pathways has been graphically represented in Figure 6B. It indicates that long-term heat stress reduces the expression of genes related to various signaling pathways, transcription factors, protein folding machinery, and abiotic stress-related genes, thus leading to reduced tolerance capacity that further affects the other metabolic processes, yield-related parameters, and quality.

Overall, continuous day stress has reduced grain fill duration, seed dimensions, yield, and TKW and increased LMW proteins (gliadins and LMW-GS). Further reduction in these parameters was observed in day–night stress which might be due to suppression of most genes and reduced metabolism, thus leading to a reduction in starch deposition and early senescence (Figures 6A,B).

## Conclusion

The present study observed the high expression of the genes related to starch biosynthesis pathway, starch transporters, transcription factors, stress response, and storage protein synthesis throughout the development under normal conditions. The expression of heat response genes increased under heat stress, which triggered regulatory, biosynthetic, and transport pathway genes to express earlier. The expression of these genes was high until 14 DAA under day stress and 7 DAA under day–night stress, which indicated that the plants tried to speed up the starch and protein synthesis and accumulation process. However, the continuous heat stress caused early senescence and maturation of the plant because of a reduction in gene expression related to antioxidant, defense, cell division, and biosynthetic machinery. The reduction in gene expression occurred because of an interplay of multiple pathways that led to a reduction in photosynthesis, glycolysis, other metabolite biosynthesis, and improper protein folding. Overall, the defense response to the day heat stress caused early gene expression, and day–night heat stress caused suppression of gene expression by activating multiple pathways, which ultimately led to the reduction in grain-filling duration, grain weight, yield, and processing quality.

## Data Availability Statement

The original contributions presented in the study are included in the article/Supplementary Material, further inquiries can be directed to the corresponding author/s.

## Author Contributions

VC and MG planned and designed the study. VC wrote the manuscript. VC, AK, and ShK performed the experiments. AmK, SS, PS, and PK helped in experiments. SS, NS, SaK, AnK, JK, and JR helped in editing. MG supervised the experiments and edited the manuscript. All authors contributed to the article and approved the submitted version.

## Conflict of Interest

The authors declare that the research was conducted in the absence of any commercial or financial relationships that could be construed as a potential conflict of interest.
