# Supplementary material for: Gene Expression and Proteomics Studies Suggest an Involvement of Multiple Pathways Under Day and Day–Night Combined Heat Stresses During Grain Filling in Wheat
Source: Front Plant Sci. 2021 May 31;12:660446. doi: 10.3389/fpls.2021.660446 (PMC8200777; doi:10.3389/fpls.2021.660446)
Supplement: Supplementary file 1 [file Data_Sheet_1.docx]

**Supplementary information**

SupplementaryTable S1 Variation in time and temperatures under controlled experiments.

| **S.No** | **Time** | **Control (24**°C**/17**°C**)** | **Day stress (35**°C**/17**°C**)** | **Combined Day-night stress (35**°C**/24**°C**)** |
| --- | --- | --- | --- | --- |
| 1. | 5:00 A.M. | 17°C | 17°C | 24°C |
| 1. | 6:00 A.M. | 24°C | 24°C | 24°C |
| 2. | 7:00 A.M. | 24°C | 27°C | 27°C |
| 3. | 8:00 A.M. | 24°C | 29°C | 29°C |
| 4. | 9:00 A.M. | 24°C | 31°C | 31°C |
| 5. | 10:00 A.M. | 24°C | 33°C | 33°C |
| 6. | 11:00 A.M. | 24°C | 35°C | 35°C |
| 7. | 5:00 P.M. | 24°C | 33°C | 33°C |
| 8. | 6:00 P.M. | 24°C | 31°C | 31°C |
| 9. | 7:00 P.M. | 24°C | 29°C | 29°C |
| 10. | 8:00 P.M. | 24°C | 27°C | 27°C |
| 11. | 9:00 P.M. | 24°C | 24°C | 24°C |
| 12. | 10:00 P.M. | 17°C | 17°C | 24°C |

Supplementary Table S2. List of genes and primer sequences utilized under study.

| **S.No** | **Gene Primer** | **Gene ID** | **Sequence (5’-3’)** | **Function** | **Reference** |
| --- | --- | --- | --- | --- | --- |
| **Starch metabolism genes** | | | | | |
| 1 | AGPL1 | Z21969.1 | Forward: GATGAGATCTCGAGGCTGATGTCC  Reverse: TACACGACGGTGCCGTCCTTGAT | (ADP glucose pyrophosphorylase,large subunit) Synthesis of ADP-glucose | Singh et al., 2015 |
| 2 | AGPS1 | X66080.1 | Forward: GCAAGATACACCATTCAGTAGTTGGAC  Reverse: GACTGTTCCACTAGGGAGTAAAGCATC | (ADP glucose pyrophosphorylase,small subunit) Synthesis of ADP-glucose | Singh et al., 2015 |
| 3 | GBSSI | AB019623.1 | Forward: CTCGCCGCCAACTACGACGTC  Reverse: TGCTCGGGAACTTCTCCTCCAC | (Granule bound starch synthase) Amylose biosynthesis | Singh et al., 2015 |
| 4 | GBSSII | AF109395.1 | Forward: GTTCCAAGTAGGTTCGAGCCATGT  Reverse: AGAGCCTCCTCCCACTTCTTTGC | (Granule bound starch synthase) Amylose biosynthesis | Singh et al., 2015 |
| 5 | SSI | AJ292521.1 | Forward: GTTTCCCACAGAATAACTGCAGGTTGC  Reverse: GTATGGTCTTTCGTCATGCCTCGC | (Soluble starch synthase) Elongation of α-1,4-linked glucan chain | Singh et al., 2015 |
| 6 | SSII | EU307274.2 | Forward: GAATCAAGTTGGCGAGGATGTCGAC  Reverse: CAATCTCTTTGCCCTCTGAAACTGCAT | (Soluble starch synthase) Elongation of α-1,4-linked glucan chain | Singh et al., 2015 |
| 7 | SSIII | EU333946.2 | Forward: GACTTCTCAGGAAATGTCTCTAGCAG  Reverse: ACCAGTGCACTTATTCTGACACGGTAC | (Soluble starch synthase) Elongation of α-1,4-linked glucan chain | Singh et al., 2015 |
| 8 | SSIV | AY044844.1 | Forward: AGAGACGAGGTTGTCAGAAACAGATG Reverse: GCATCTAAAAGAGCATGCTCCTTCTC | (Soluble starch synthase) Elongation of α-1,4-linked glucan chain | Singh et al., 2015 |
| 9 | SBEI | Y12320.1 | Forward: CGCTCGACGACAAGTTTTCCTTCC  Reverse: CAAGTGCGGGGTGGAGACAGG | (Starch branching enzyme) Branching through α-1,6-linkage | Singh et al., 2015 |
| 10 | SBEIII | JQ346193.1 | Forward: AGAGAAGGGCAAGAACCAGATGAATAC  Reverse: TCTGGTTTCAGCATCAGGTATTTCACC | (Starch branching enzyme) Branching through α-1,6-linkage | Singh et al., 2015 |
| 11 | ISA1 | AF548380.1 | Forward: GTCTTCAACCATACAGCTGAGGGT  Reverse: GAACCTCTGGTCATTATGGATGCAAG | (Isoamylase) Debranching of α-1,6-linkages in amylopectin | Singh et al., 2015 |
| 12 | ISA2 | JX473824.1 | Forward: TCTGGCACGTCTCGCTGGAGA  Reverse: CTGTAGCTCGGTGGATTCGCCA | (Isoamylase) Debranching of α-1,6-linkages in amylopectin | Singh et al., 2015 |
| 13 | PUL | EF137375.1 | Forward: CTCCGCTTTTTCGTCTTAGTACAGC  Reverse: GCATCTGATGAATTCACCTGCACAG | (Pullulanase) | Singh et al., 2015 |
| 14 | Pho1 | EU595762.1 | Forward: TGGGGATCATGCCAAGGCCAATGA  Reverse: CCAGTTGAGAGAATCACCAGCCCT | (Starch phosphorylase (pho) Reversible phosphorylation of α-glucan | Singh et al., 2015 |
| 15 | Pho2 | AF275551.1 | Forward: TGGCCAGCAAAAAGCGCCTAGC  Reverse: TCTGCCTGTCTGCTGCGCTCA | (Starch phosphorylase (pho) Reversible phosphorylation of α-glucan | Singh et al., 2015 |
| 16 | DPE1 | DQ068045.1 | Forward: CGAGGGGTATGAGCAGATGATGC  Reverse: GTACTACCACAGTTTGCATCCTGTCCT | (Disproportionating enzyme) Cleavage and transfer of α-1,4-linked glucan | Singh et al., 2015 |
| 17 | AMY | X05809.1 | Forward: TGCTTTGTCTCGCCTCCAGCTTAG  Reverse: CGTACTTGGAGTTGAGGTTGTAGAGC | (α-amylase) Hydrolysis of the internal α-1,4-linked glucan | Singh et al., 2015 |
| 18 | BMY | X98504.1 | Forward: CTAGCCAACTATGTCCAAGTCTACGT  Reverse: ACTGTGGGATGGGGATGTTGACGA | (β-amylase) Hydrolysis of the second α-1,4-linked glucan | Singh et al., 2015 |
| **Starch transporters** | | | | | |
| 19 | TPT | AF314182.2 | Forward: GTATCAGATCTTTTCCTGGTGGGATTG Reverse: TACGAGTAGAGGGCAACACCAGCT | (Triose phosphate translocator) Export of triose phosphates from chloroplast to cytosol | Singh et al., 2015 |
| 20 | SUT | AF408843.1 | Forward: CCTCTCGCCATTCTGTACAGTGTC  Reverse: GCAGCAGGAATATGCCGACGATG | (Sucrose transporter) Transport of sucrose from leaf cytosol to seed cytosol | Singh et al., 2015 |
| 21 | BT1 | GQ916634 | Forward: GACCAACTACTATGCCTACGACACC Reverse: TCTTCTTGCAGGCCTCGTAGCACA | (Brittle-1 transporter) Export of ADP-glucose from cytosol to seed amyloplast | Singh et al., 2015 |
| **Regulator genes/transcription factors** | | | | | |
| 22 | *TaRSR1* | JX473823.1 | Forward: GCTGCTCAATGAAGTTGCTGCTGAAG  Reverse: GATTGGTGGATGCTCGGTCAGAAG | (Rice starch regulator (TaRSR1) Regulator of several starch metabolic genes | Singh et al., 2015 |
| 23 | SPA | Y09013.1 | Forward: AAGTTGGACGCGCATCTCGCCG  Reverse: CTGACATCTCCGGTGTGGTTTTCTGAG | Regulator of several starch metabolic genes | Singh et al., 2015 |
| 24 | RabD 54832 | AK335455.1 | Forward: ATCGGAGACTCAGGTGTTGG  Reverse: GTTCTGAAGCGTTCTTGCCC | Rab GTPases are involved in regulation fusion of transport vesicles with their different target membranes. | Tyler et al., 2015 |
| 25 | RabD 47209 | AK332302.1 | Forward: AGGTCGTCGATACAGAGGAG  Reverse: GGCTCGCCATCTTGTTCTTG | Rab GTPases are involved in regulation fusion of transport vesicles with their different target membranes. | Tyler et al., 2015 |
| 26 | TaC4ZFP | KJ685918.1 | Forward: GAATGAGGAGCTTGGCTTGAA  Reverse: CAGGCTACAACACTGAGCACAAA | C4-type zinc finger transcription factor (TaC4ZFP) | Xue et al., 2015 |
| **Stress responsive genes** | | | | | |
| 27 | HSP70 | AF005993.1 | Forward: CTTCGTCCAGGAGTTCAAGC  Reverse: GTCGATCTCGATGGTGGTTT | Heat Shock protein- role in protein folding | Kumar et al., 2014 |
| 28 | CDPK | EF068170.1 | Forward: TGCCATTCACTTGGTGTGAT  Reverse: GGCCTGGCTTGAAGTAGATG | Calcium dependent protein kinase -signalling molecule | Kumar et al., 2014 |
| 29 | HSP90 | JN052206.1 | Forward: TGAGTATGGGTGGACTGCCAACAT  Reverse: TCTCGAAGAGCAGCATCACAAGGT | Heat Shock protein | Kumar et al., 2014 |
| 30 | HSP26 | HM802264.1 | Forward: ATCGACGTGAAGGTCCAGTGATGA  Reverse: AGAAAGGATCGGAGAAGAACGGCA | Heat Shock protein | Kumar et al., 2013 |
| 31 | CAT | GU984379.1 | Forward: CAAGAGCGATTCATCAACAGAT  Reverse: AGACCAGTAGGAGAGCCAGATG | Catalase | Kumar et al., 2014 |
| 32 | Can3174 | KC788219.1 | Forward: GGCCGGTCATTCATAGAGAA  Reverse: TGAATTCGGACCAAAAGGAG | Hypothetical protein candidate 3174 | Kumar et al., 2014 |
| 33 | RCA | AF251264.1 | Forward: CGCAAGTACGACTTCGACAA  Reverse: CAGGATGAGAGGGACCTTGA | Rubisco activase | Kumar et al., 2014 |
| 34 | GAPDH | EF592180.1 | Forward: CGGAAAGTTGACTGGAATGG  Reverse: ATCATAGGTTGCTGGCTTCG | Glyceraldehyde-3-phosphate dehydrogenase | Kumar et al., 2014 |
| 35 | SOD | JQ613154.1 | Forward: TCCTTTGACTGGCCCTAATG  Reverse: CTTCCACCAGCATTTCCAGT | Superoxide dismutase | Kumar et al., 2013 |
| 36 | dreb | KF737910.1 | Forward: CCACCCATAGAATCAAGAAAGAG  Reverse: GCAAATTACCCAATCCTGAC | Dehydration responsive element binding proteins (DREBs) Transcription factor that induces the expression of a large number of functional genes and imparts tress endurance to plants. | Hassan et al., 2015 |
| **Storage proteins** | | | | | |
| 37 | ω Glia QF18 | AF280605.1 | Forward: AAGGCAAGCAAGCAGTAG  Reverse: GATTGTTGAGGTGATTGTAGC | Seed Storage protein | Altenbach and Kothari 2007 |
| 38 | ω Glia QF21 | AB181300.1 | Forward: CAACCACCACAACAATTC  Reverse: TTACATCTCTTCATTTCATAGG | Seed Storage protein | Altenbach and Kothari 2007 |
| 39 | γ Glia QF69 | AY338386.1 | Forward: GATCCTGCGGCCACTATTTCAGCTC  Reverse: CAGGTGGCACATACACGTTGCACAT | Seed Storage protein | Altenbach and Kothari 2007 |
| 40 | Pre-α/β-gliadin | K03076.1 | Forward: GACCTTTCTCATCCTTGTCCTCCT  Reverse: CTGTGAATATGGTAGTTGCGGCTG | alpha/beta-gliadin precursor mRNA | Singh et al., 2014 |
| 41 | γ-Gliadin | FJ006618.1 | Forward: TCTCTACAACAACAGATGAACCCCTG  Reverse: GCCTTGTTGTTGTTCTTGCTGCATG | Gamma gliadin - seed storage protein | Singh et al., 2014 |
| 42 | LMW -METSHIP7 | KR612277.1 | Forward: TGGTTTGGAGAGACCATCG  Reverse: GCTGCTGGAGGAATACTTTGC | Low molecular weight gluteniun subunit | Altenbach 1998 |
| 43 | LMW -MET F2 | KR612308.1 | Forward: AGACCTTCCTCGTCTTTGCC  Reverse: GTTGGTAGAGACCTTGAACCG | Low molecular weight gluteniun subunit | Altenbach 1998 |
| 44 | Glu-tritprt 3 | AJ567980.1 | Forward: CCTCTTTGCGGCAGTAGTYGT  Reverse: CTCGCRCTCACABTGTAGTTG | Seed storage protein | Zeltner et al., 2009 |
| 45 | γ gliadin He | HE819390.1 | Forward: ACAGCTGCCAGTTGAAGCG  Reverse: GACGATGGCATGCACAATGT | Seed storage protein | Wan et al., 2013 |

Supplementary Table S3. Gene expression (2^-ΔΔCt^) values determined by qRT-PCR

|  | 7DAA | | | 14DAA | | | 21DAA | | | 28DAA | | |
| --- | --- | --- | --- | --- | --- | --- | --- | --- | --- | --- | --- | --- |
|  | control | day stress | day-night stress | control | day stress | day-night stress | control | day stress | day-night stress | control | day stress | day-night stress |
| AGPSS | 0.74 | 2.55 | 0.36 | 4.30 | 1.42 | 1.74 | 5.12 | 2.07 | 1.69 | 9.70 | 7.71 | 5.84 |
| AGPLS | 1.02 | 4.08 | 1.45 | 17.67 | 1.75 | 0.39 | 14.79 | 0.51 | 1.33 | 11.57 | 1.43 | 1.40 |
| GBSSI | 0.96 | 4.09 | 0.95 | 1.75 | 0.93 | 0.73 | 2.50 | 0.80 | 1.77 | 6.71 | 1.08 | 2.51 |
| GBSSII | 1.02 | 3.11 | 1.11 | 0.76 | 0.89 | 0.83 | 0.80 | 0.77 | 1.32 | 4.51 | 1.26 | 1.17 |
| SS1 | 1.23 | 2.54 | 0.92 | 1.48 | 0.80 | 0.66 | 1.16 | 0.53 | 2.05 | 5.20 | 1.87 | 1.52 |
| SS2 | 1.22 | 0.96 | 2.46 | 2.22 | 1.52 | 0.02 | 1.80 | 0.10 | 0.10 | 8.72 | 0.14 | 0.13 |
| SS3 | 0.66 | 0.79 | 0.36 | 0.29 | 0.23 | 0.23 | 0.30 | 0.24 | 0.70 | 1.72 | 0.25 | 0.44 |
| SS4 | 1.03 | 0.75 | 0.68 | 4.52 | 3.35 | 0.85 | 0.50 | 0.88 | 0.95 | 0.53 | 0.89 | 0.85 |
| SBE1 | 0.79 | 0.51 | 0.84 | 5.03 | 2.59 | 0.43 | 0.48 | 0.85 | 0.74 | 0.57 | 1.18 | 0.82 |
| SBE3 | 1.02 | 7.19 | 4.23 | 7.70 | 3.48 | 1.03 | 0.75 | 1.40 | 1.02 | 0.43 | 2.60 | 1.76 |
| ISA1 | 0.87 | 1.99 | 1.19 | 3.93 | 3.25 | 0.94 | 0.78 | 1.00 | 0.96 | 1.18 | 1.89 | 1.44 |
| ISA2 | 0.91 | 0.69 | 0.60 | 3.54 | 2.95 | 0.73 | 0.39 | 0.60 | 0.86 | 0.53 | 0.84 | 0.62 |
| PUL | 0.96 | 2.92 | 4.08 | 4.36 | 4.92 | 0.80 | 0.37 | 1.29 | 2.56 | 1.52 | 1.11 | 2.55 |
| Pho1 | 0.95 | 3.00 | 1.05 | 4.59 | 2.19 | 0.49 | 0.42 | 1.98 | 1.31 | 0.62 | 1.25 | 1.76 |
| Pho2 | 0.84 | 1.33 | 0.67 | 1.45 | 2.23 | 1.08 | 0.01 | 0.29 | 0.89 | 0.02 | 0.14 | 0.22 |
| DPE-I | 0.98 | 0.75 | 0.70 | 0.57 | 4.62 | 1.41 | 0.01 | 0.12 | 0.84 | 0.02 | 0.10 | 0.16 |
| AMY | 1.20 | 3.37 | 0.27 | 114.77 | 0.43 | 0.10 | 0.09 | 0.08 | 0.29 | 0.64 | 1.25 | 0.30 |
| BMY | 1.71 | 60.30 | 5.80 | 74.04 | 69.01 | 1.57 | 846.96 | 0.66 | 2.43 | 23.28 | 1.81 | 2.00 |
| TPT | 0.80 | 1.65 | 0.98 | 5.43 | 2.19 | 0.80 | 0.43 | 0.59 | 0.75 | 0.40 | 0.77 | 0.53 |
| SUT | 0.88 | 3.84 | 1.09 | 5.14 | 1.79 | 0.46 | 0.35 | 0.95 | 0.90 | 0.43 | 1.23 | 1.11 |
| BT1 | 0.97 | 1.38 | 0.72 | 4.18 | 2.13 | 0.63 | 1.17 | 1.72 | 0.99 | 1.43 | 2.33 | 1.84 |
| Ta RSR1 | 1.03 | 1.40 | 2.39 | 5.76 | 2.83 | 0.72 | 0.37 | 1.33 | 0.90 | 0.62 | 1.13 | 1.28 |
| SPA (OsbZIP58) | 1.02 | 3.67 | 0.94 | 0.91 | 0.96 | 0.77 | 0.92 | 0.68 | 1.05 | 5.35 | 0.89 | 0.93 |
| RabD 47209 | 0.98 | 1.97 | 0.85 | 3.87 | 2.84 | 0.35 | 0.46 | 0.87 | 0.54 | 0.16 | 1.60 | 1.81 |
| RabD 54832 | 0.98 | 1.98 | 1.09 | 7.22 | 2.68 | 0.51 | 0.17 | 0.34 | 0.85 | 0.28 | 0.41 | 0.48 |
| TaC4ZP | 0.98 | 2.70 | 1.46 | 11.46 | 1.10 | 0.79 | 0.53 | 2.07 | 0.48 | 0.43 | 0.89 | 3.60 |
| HSP26 | 0.98 | 2.47 | 0.86 | 1.25 | 0.42 | 0.10 | 1.08 | 0.24 | 1.98 | 3.39 | 2.60 | 3.08 |
| HSP70 | 1.68 | 95.61 | 38.01 | 38.14 | 5.18 | 0.69 | 11.34 | 0.29 | 0.70 | 10.94 | 0.96 | 0.89 |
| HSP90 | 1.03 | 17.70 | 2.61 | 20.10 | 4.30 | 0.62 | 7.74 | 0.42 | 1.46 | 18.50 | 0.95 | 1.36 |
| CDPK | 1.65 | 13.26 | 1.74 | 25.23 | 3.92 | 1.45 | 59.05 | 0.89 | 1.35 | 20.59 | 2.32 | 2.39 |
| CAT | 0.95 | 29.29 | 9.74 | 11.83 | 0.90 | 1.08 | 4.84 | 1.08 | 2.30 | 34.98 | 4.84 | 4.01 |
| Can 3174 | 1.05 | 5.42 | 1.52 | 4.22 | 1.61 | 0.56 | 0.08 | 0.70 | 3.48 | 4.33 | 5.38 | 5.65 |
| GAPDH | 0.84 | 89.62 | 20.08 | 117.30 | 1.09 | 0.80 | 8.59 | 1.43 | 1.23 | 10.21 | 1.23 | 1.55 |
| SOD | 0.69 | 1.76 | 0.53 | 13.90 | 0.50 | 0.79 | 1.21 | 1.65 | 2.51 | 6.13 | 0.85 | 1.53 |
| dreb | 1.09 | 3.74 | 0.94 | 1.91 | 1.10 | 0.62 | 1.26 | 0.36 | 1.77 | 5.71 | 1.64 | 1.64 |
| RCA | 0.84 | 0.72 | 0.67 | 4.31 | 2.25 | 0.59 | 0.42 | 0.60 | 0.89 | 0.51 | 0.84 | 0.91 |
| Pre alpha/beta gliadin | 0.95 | 2.24 | 1.64 | 9.85 | 102.15 | 0.51 | 136.25 | 0.40 | 1.74 | 42.87 | 1.35 | 1.30 |
| gamma- glia QF 69 | 3.01 | 102.29 | 30.76 | 466.26 | 28.70 | 2.27 | 724.19 | 0.15 | 0.34 | 59.17 | 0.54 | 0.39 |
| gamma-gliadin | 0.96 | 3.12 | 1.16 | 1.94 | 1.64 | 0.81 | 14.74 | 0.65 | 1.29 | 8.07 | 1.13 | 1.41 |
| gamma- gliadin- He | 0.75 | 2.86 | 7.99 | 62.41 | 2.27 | 1.18 | 0.08 | 0.21 | 0.21 | 0.19 | 0.30 | 0.29 |
| Omega glia QF 18 | 0.97 | 16.86 | 55.53 | 20.53 | 2.19 | 8.26 | 0.18 | 0.05 | 1.14 | 0.11 | 0.13 | 0.08 |
| Omega glia QF 21 | 0.95 | 16.41 | 66.64 | 25.75 | 3.08 | 7.80 | 0.21 | 0.06 | 1.34 | 0.16 | 0.08 | 0.07 |
| LMW Metship 7 | 0.95 | 0.71 | 1.17 | 0.77 | 23.88 | 0.62 | 31.00 | 2.01 | 1.18 | 0.94 | 2.61 | 0.50 |
| LMW Metship F2 | 0.99 | 0.44 | 0.60 | 4.67 | 1.56 | 0.31 | 0.09 | 0.19 | 0.60 | 0.12 | 0.30 | 0.23 |
| glutritprt 3 | 1.02 | 2.26 | 4.59 | 8.04 | 3.51 | 0.60 | 0.13 | 0.26 | 1.31 | 0.00 | 0.00 | 0.00 |

Supplementary Table S4 Concentration of different protein fractions analysed by 2D-quant kit from different temperature regimes

| **S.No** | **Conditions** | **Albumins (µg/µl)** | **Gliadins (µg/µl)** | **Glutenins (µg/µl)** | **Total Protein (µg/µl)** | **Glutenin/gliadin ratio** |
| --- | --- | --- | --- | --- | --- | --- |
| 1. | 24/17 | 0.95±0.02^a^ | 1.04±0.03^a^ | 1.66±0.01^c^ | 3.65 | 1.60 |
| 2. | 35/17 | 1.37±0.03^c^ | 1.57±0.06^c^ | 1.53±0.02^b^ | 4.47 | 0.98 |
| 3. | 35/24 | 1.11±0.01^b^ | 1.26±0.02^b^ | 1.28±0.02^a^ | 3.65 | 1.02 |
